# Hybrid EMT Phenotype and Cell Membrane Tension Promote Colorectal Cancer Resistance to Ferroptosis

**DOI:** 10.1002/advs.202413882

**Published:** 2025-02-22

**Authors:** Xiaowei Wei, Yutong Ge, Yaolin Zheng, Sunyan Zhao, Yuhan Zhou, Yuhan Chang, Nuofan Wang, Xiumei Wang, Juan Zhang, Xuanchang Zhang, Liqiao Hu, Youhua Tan, Qiong Jia

**Affiliations:** ^1^ Department of Oncology Nanjing First Hospital Nanjing Medical University Nanjing 210006 China; ^2^ Department of Oncology The First Affiliated Hospital of Nanjing Medical University Nanjing 210029 China; ^3^ Department of Respiratory Critical Care and Sleep Medicine Xiang'an Hospital of Xiamen University School of Medicine Xiamen University Xiamen 361102 China; ^4^ Cancer Center Zhongshan Hospital Fudan University Shanghai 200032 China; ^5^ School of Medicine Southeast University Nanjing 210009 China; ^6^ Guangzhou National Laboratory Guangzhou 510005 China; ^7^ The Hong Kong Polytechnic University Shenzhen Research Institute Shenzhen 518000 China; ^8^ Department of Biomedical Engineering The Hong Kong Polytechnic University Hong Kong 999077 China

**Keywords:** colorectal cancer, ferroptosis, hybrid EMT, membrane tension, physical environment

## Abstract

Intratumoral heterogeneity, including epithelial–mesenchymal transition (EMT), is one major cause of therapeutic resistance. The induction of ferroptosis, an iron‐dependent death, has the potential in overcoming this resistance to traditional treatment modalities. However, the roles of distinct EMT phenotypes in ferroptosis remain an enigma. This study reports that 3D soft fibrin microenvironment confers colorectal cancer (CRC) cells hybrid EMT phenotype and high level of resistance to ferroptosis. The activation of histone acetylation and WNT/β‐catenin signaling drives this EMT phenotypic transition, which promotes the defense of 3D CRCs against ferroptosis via glutathione peroxidases/ferritin signaling axis. Unexpectedly, E‐cadherin knockout in 3D but not 2D CRCs mediates an integrin β_3_ marked‐late hybrid EMT state and further enhances the resistance to ferroptosis via integrin‐mediated tension and mitochondrial reprogramming. The inhibition of integrin α_v_β_3_‐mediated tension and WNT/β‐catenin‐mediated hybrid EMT sensitizes 3D CRCs with and without E‐cadherin deficiency to ferroptosis in vivo, respectively. Further, the EMT phenotype of patient‐derived tumoroids is associated with CRC therapeutic resistance. In summary, this study uncovers previously unappreciated roles of hybrid EMT and cell membrane tension in ferroptosis, which not only predict the treatment efficacy but also potentiate the development of new ferroptosis‐based targeted therapeutic strategies.

## Graphical abstract

Colorectal cancer cells (CRCs) cultured in 3D soft fibrin exhibit hybrid epithelial–mesenchymal transition (EMT) mediated ferroptosis resistance via WNT/β‐catenin signaling. Unlike 2D cells, 3D CRCs exhibit reduced E‐cadherin, knockout of which further enhances the defense against ferroptosis via adhesion‐mediated tension. This work reveals the distinct roles of EMT states in CRC ferroptosis and the biomechanical vulnerability of therapeutic resistance.

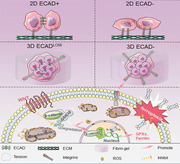

## 1 Introduction

The mainstream anticancer treatments, such as chemotherapy and radiotherapy, kill tumor cells through the induction of apoptosis, necrosis, and the latest proposed ferroptosis.^[^ ^1^ ^]^ However, the efficacy is often plagued by therapeutic resistance, which could arise from the diversity of tumor cells or intratumoral heterogeneity, one hallmark of cancer.^[^ ^2^ ^]^ Despite genomic variations, the robust transition among different phenotypic states mediates a high level of tumor cell plasticity and heterogeneity, which can be altered upon tumor evolution, cancer therapy, and microenvironmental changes.^[^ ^3^ ^]^ This evolving alteration poses challenges to the targeting of therapy‐resistant phenotypes.

As an important cause of heterogeneity, epithelial–mesenchymal transition (EMT) involves the conversion from epithelial to mesenchymal phenotype and plays critical roles in tumorigenesis, progression, and therapeutic resistance.^[^ ^5^ ^]^ Previous studies suggest that EMT is not a binary process, but instead involves different states that can be dynamically interconvertible among each other, such as fully epithelial, partial EMT, hybrid EMT, and fully mesenchymal states.^[^ ^6^, ^7^, ^8^ ^]^ The phenotypic evolution during EMT depends on a variety of mechanisms, such as TGF‐SMAD, canonical or noncanonical WNT, receptor tyrosine kinase, and ECM–integrin signaling pathways, which can be activated by various stimuli from the dynamically evolving local microenvironment.^[^ ^9^ ^]^ Remarkably, different EMT phenotypes exhibit distinct therapeutic resistance to cancer treatment. For instance, breast cancer cells with a fully mesenchymal state are more chemoresistant than epithelial or partial EMT cells.^[^ ^10^ ^]^

The addiction to iron renders cancer cells vulnerable to ferroptosis, a form of recently discovered cell death driven by iron‐dependent lipid peroxidation,^[^ ^11^, ^12^ ^]^ and thus, the induction of ferroptosis could be one promising strategy to sensitize cancer cells to chemoradiotherapy and immunotherapy^[^ ^13^, ^14^, ^15^ ^]^ and circumvents drug resistance.^[^ ^16^ ^]^ Notably, EMT phenotype is associated with the level of defense against ferroptosis. For example, carcinomas with mesenchymal or metastatic properties exhibit increased sensitivity to ferroptosis;^[^ ^17^, ^18^ ^]^ rather, the EMT transcription factor slug protects glioblastoma cells from ferroptosis by reversing SLC7A11.^[^ ^19^ ^]^ Nevertheless, the relationship between different EMT phenotypes and ferroptosis remains poorly understood, which poses a challenge to the overcoming of therapeutic resistance arising from EMT‐mediated heterogeneity.

Further, the physical microenvironment critically influences various tumor cell functions, including EMT phenotype and ferroptosis. Matrix stiffness has been found to induce EMT in pancreatic^[^ ^20^ ^]^ and breast^[^ ^21^ ^]^ cancer cells; nanostructured architectures facilitate the conversion of breast and prostate cancer cells from mesenchymal to epithelial phenotype;^[^ ^22^ ^]^ stiff matrix promotes the sensitivity of hepatocellular carcinoma cells to ferroptosis via mechanotransduction‐mediated YAP activation and cell membrane permeabilization.^[^ ^23^ ^]^ However, it remains unclear whether the physical tumor microenvironment can regulate tumor cell ferroptosis by impacting their EMT state.

Our previous work has shown that 3D soft fibrin gels can select tumor‐repopulating cells (TRCs) with high tumorigenic and metastatic potential,^[^ ^24^ ^]^ which is through the mechanotransduction‐mediated histone demethylation and Sox2 upregulation.^[^ ^25^, ^26^ ^]^ The antiapoptosis ability of TRCs depends on cytoskeletal tension.^[^ ^27^ ^]^ Our latest study shows that 3D soft fibrin downregulates capping protein inhibiting regulator of actin dynamics (CRAD) in colorectal cancer cells (CRCs), which further decreases YAP activity and promotes the transcription of NANOG and OCT4, leading to cancer stemness and metastasis.^[^ ^28^, ^29^ ^]^ Interestingly, 3D soft fibrin not only enhances the malignancy of CRCs,^[^ ^29^ ^]^ including the resistance to chemotherapy,^[^ ^24^ ^]^ but also transforms tumor cells into a hybrid EMT phenotype, indicating the association between EMT state and therapeutic resistance. To further explore this relationship, the current study reported that CRCs selected by 3D soft fibrin exhibited a hybrid EMT state, which conferred tumor cells with the ability to evade ferroptosis. In contrast to 2D cells, 3D CRCs exhibited low E‐cadherin (about half of that in 2D cells), knockout of which mediated a CD61 (integrin β_3_) labeled‐late hybrid EMT phenotype and further enhanced the defense against ferroptosis via the adhesion‐mediated tension. This work revealed the distinct roles of EMT states in CRC ferroptosis and the biomechanical vulnerability of therapeutic resistance, which might provide new targets for CRC treatment.

## 2 Results

### 2.1 3D CRCs Are Highly Resistant to Ferroptosis

**Figure 1:**
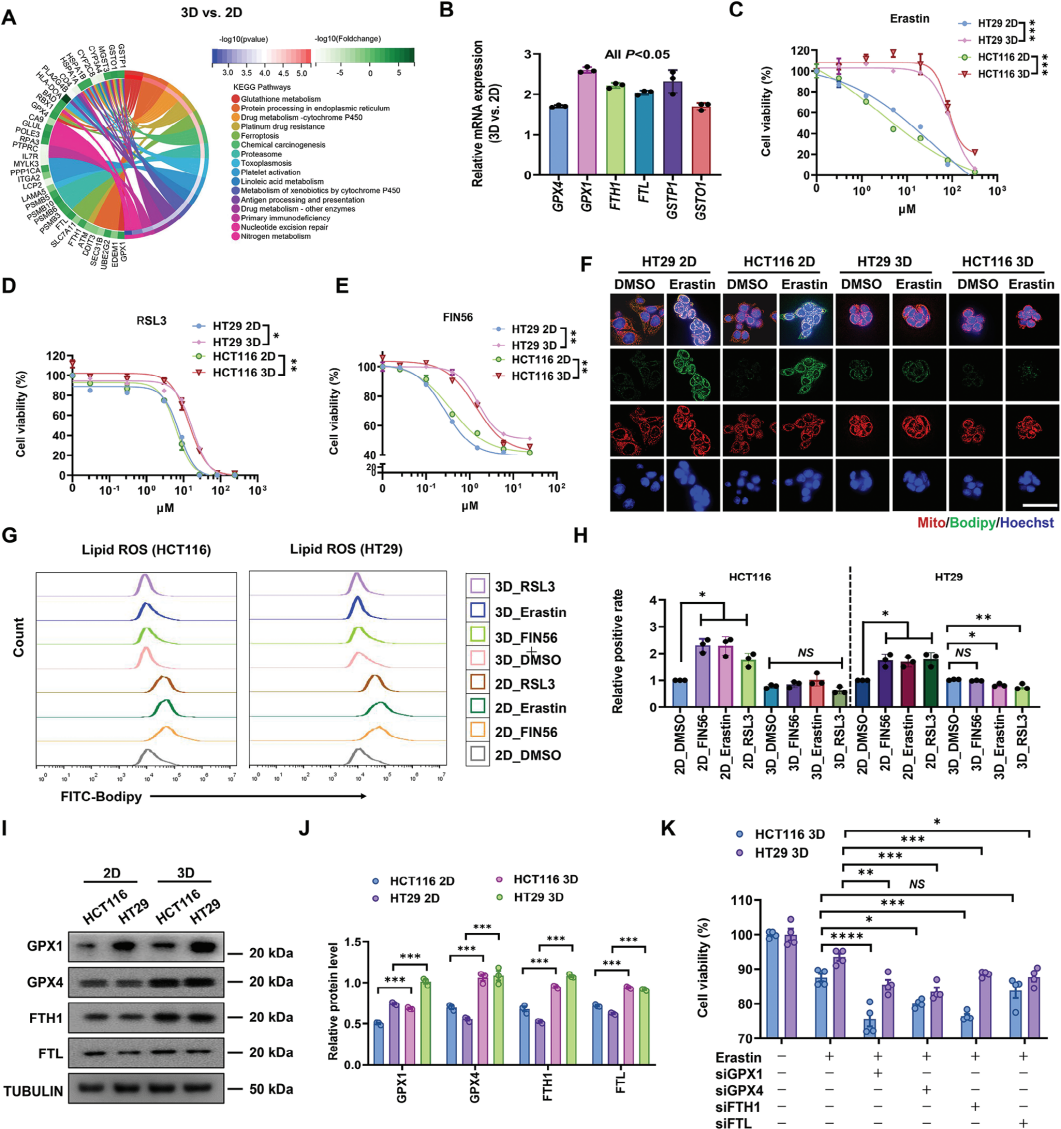
3D CRCs are highly resistant to ferroptosis. A) The functional enrichment analysis of differential gene sets of 2D and 3D CRCs. R software package clusterProfiler (version 3.14.3) was used to obtain the results of gene set enrichment. The minimum gene set was set to 5 and the maximum gene set was set to 5000, with a *P* value of <0.05, which were considered statistically significant. 2D, cells cultured on rigid plastic dishes for 3 days; 3D, cells cultured in 90‐Pa 3D fibrin gels for 3 days. *n* = 3. B) HCT116 cells were cultured on 2D or in 3D for 3 days. Total mRNA was extracted, and qPCR was performed to detect the expression of indicated genes in 3D cells relative to 2D cells (set to 1). Means ± SEMs; *n* = 3. *P* < 0.05 for all the indicated 3D versus 2D differentially expressed genes analyzed by two‐tailed Student's *t*‐test. **C**. Relative cell viability of 2D or 3D CRCs (including HCT116 and HT29) treated with DMSO or different concentrations of Erastin (0.3125, 1.25, 5, 20, 80, and 320 µm) for 24 h. Means ± SEMs; *n* = 4. ***P < 0.001 analyzed by two‐tailed Student's *t*‐test. D) Relative cell viability of 3D or 2D CRCs treated with DMSO or different concentrations of RSL3 (0.03, 0.3, 3, 9, 27, 81, and 243 µm) for 24 h. Means ± SEMs; *n* = 4. *P < 0.05, **P < 0.01 analyzed by two‐tailed Student's *t*‐test. E) Relative cell viability of 3D or 2D CRCs treated with DMSO or different concentrations of FIN56 (0.025, 0.1, 0.4, 1.5, 6, 24 µm) for 24 h. Means ± SEMs; *n* = 4. **P < 0.01 analyzed by two‐tailed Student's *t*‐test. F) 2D and 3D CRCs were treated with DMSO or 15 µm Erastin for 24 h, and stained with a BODIPY‐C11 probe (green), Mitotracker (red), or Hoechst (blue), respectively. Scale bar: 40 µm. Images were captured by ELyra 7 with Lattice SIM. At least three views were randomly selected for each condition. G,H) 2D and 3D CRCs were treated with 3 µm RSL3, 15 µm Erastin, 3 µm FIN56, or DMSO for 24 h, and then lipid ROS was measured using the BODIPY‐C11 probe by flow cytometry. Means ± SEMs; *n* = 3. *P < 0.05, **P < 0.01. NS, nonsignificant difference among all the indicated groups analyzed by the ordinary one‐way ANOVA with Tukey's multiple comparison test. I,J) GPX1, GPX4, FTH1, FTL, and TUBULIN protein levels in 2D and 3D CRCs cultured for 3 days were detected by western blotting. Relative protein levels of each protein were normalized to TUBULIN. Means ± SEMs; *n* = 3. ***P < 0.001 analyzed by two‐tailed Student's *t*‐test. K) CRCs were transfected with negative control or siRNAs targeting *GPX1*, *GPX4*, *FTH1* or *FTL*, and then cultured in 90‐Pa 3D fibrin gels for 24 h. Relative cell viability of cells treated with 15 µm Erastin for 24 h was detected. Means ± SEMs; *n* = 4. NS, nonsignificant difference, *P < 0.05, **P < 0.01, ***P < 0.001, ****P < 0.0001 analyzed by the ordinary one‐way ANOVA with Tukey's multiple comparison test.

Our recent studies have shown that CRCs after culture in 3D soft fibrin are highly malignant.^[^ ^29^ ^]^ Latest evidence suggests that ferroptosis, a distinct iron‐dependent type of cell death, plays important roles in tumor progression.^[^ ^1^, ^12^ ^]^ Ferroptosis is primarily caused by unrestricted phospholipid peroxidation, a process that occurs largely on the basis of the metabolites reactive oxygen species (ROS), phospholipids containing polyunsaturated fatty acid chains (PUFA‐PL), and increased iron accumulation, and thus involves the dysregulations in multiple metabolic and antioxidant systems centered around glutathione (GSH).^[^ ^16^, ^30^, ^31^ ^]^ Yet, the link between the malignancy of 3D‐cultured CRCs and ferroptosis remains poorly understood. To address this issue, we conducted RNA sequencing (RNA‐Seq) and KEGG analysis of 2D‐ and 3D‐cultured CRCs, showing clear differences in the pathways related to glutathione metabolism, ferroptosis, and drug metabolism (**Figure** **1**A). 3D CRCs upregulated the key regulators of ferroptosis and glutathione metabolism (*GPX4*, *GPX1*, *FTH1*, *FTL*, *GSTP1*, *GSTO1*) (Figure 1B), indicating the potential resistance to ferroptosis. To test this possibility, we treated two 2D‐ and 3D‐cultured CRC lines (HCT116 and HT29) with three commonly used ferroptosis inducers (FINs), including Erastin (FIN class I), RSL3 (FIN class II), and FIN56 (FIN class III). The results showed that 3D cells were markedly resistant to ferroptosis (Figure 1C–E). Under FIN treatments, 2D cells produced a large amount of lipid peroxides (lipid ROS) and ferrous ions, whereas 3D cells generated higher GSH and γ‐glutamylcysteine but did not increase the levels of lipid ROS and ferrous ions (Figure S1A–D, Supporting Information; Figure 1F–H). Further, after FIN induction, the atrophy of mitochondria and reduction or even disappearance of mitochondrial cristae were clearly visualized under transmission electron microscopy (Figure S1E, Supporting Information), confirming the occurrence of ferroptosis. Cell viability under Erastin treatment could be rescued by the inhibition of ferroptosis only but not apoptosis, necrosis, or autophagy (Figure S1F, Supporting Information). We further found the upregulation of glutathione metabolism‐associated molecules glutathione peroxidase 1/4 (GPX1/GPX4)^[^ ^32^, ^33^ ^]^ and iron metabolism‐associated molecules ferritin heavy chain 1 (FTH1) and ferritin light chain (FTL)^[^ ^34^ ^]^ at the protein level in 3D‐cultured CRCs (Figure 1I,J). Silencing these proteins attenuated the defense against ferroptosis (Figure 1K; Figure S1G, Supporting Information). Collectively, these results suggest that 3D‐cultured CRCs are highly reluctant to ferroptosis.

### 2.2 3D CRCs Undergo Histone Acetylation‐Mediated Hybrid EMT

**Figure 2:**
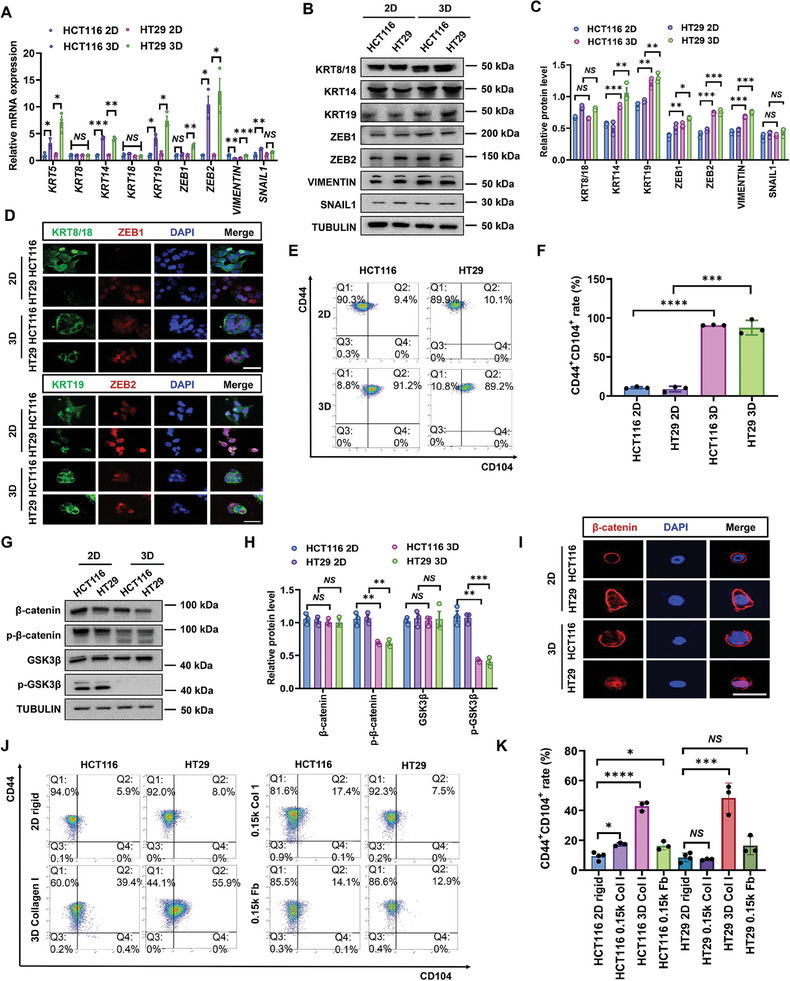
3D CRCs undergo hybrid EMT. A) Total mRNA was extracted to detect the expression of epithelial and mesenchymal markers of 2D and 3D CRCs. Means ± SEMs; *n* = 3. NS, nonsignificant difference, *P < 0.05, **P < 0.01, ***P < 0.001 analyzed by two‐tailed Student's *t*‐test. B,C) Western blotting analysis of 2D and 3D CRCs for KRT8/18, KRT19, KRT14, VIMENTIN, ZEB1, ZEB2, SNAIL1, and TUBULIN. Relative protein levels of each protein were normalized to TUBULIN. Means ± SEMs; *n* = 3. NS, nonsignificant difference, **P < 0.01, ***P < 0.001 analyzed by two‐tailed Student's *t*‐test. D) Cells were fixed and stained with KRT8/18 (green) and ZEB1 (red), KRT19 (green), and ZEB2 (red) antibodies. Nuclei were visualized by DAPI (blue). Scale bar: 50 µm. At least three views were randomly selected for each condition. E,F) Flow cytometry profiles for CD104 and CD44 of 2D and 3D CRCs. Means ± SEMs; *n* = 3. ***P < 0.001, ****P < 0.0001 analyzed by two‐tailed Student's *t*‐test. G,H) Phospho‐β‐catenin, total β‐catenin, phospho‐GSK3β, total GSK3β, and TUBULIN protein levels in 2D and 3D CRCs cultured for 3 days were detected by western blotting. Relative protein levels of each protein were normalized to TUBULIN. Means ± SEMs; *n* = 3. NS, nonsignificant difference, **P < 0.01, ***P < 0.001 analyzed by the ordinary one‐way ANOVA with Tukey's multiple comparison test. I) Cells were cultured for 10 h and then stained by β‐catenin (red) showing differential subcellular localization. Nuclei were visualized by DAPI (blue). Scale bar: 30 µm. At least three views were randomly selected for each group. J,K) Flow cytometry profiles for CD44/CD104 of cells cultured on 2D rigid plastic dishes, 0.15 kPa PA gels coated with Fibrinogen (Fb) or Collagen I (Co1), or cultured in 3D Collagen gels. Means ± SEMs; *n* = 3. NS, nonsignificant difference, *P < 0.05, ***P < 0.001, ****P < 0.0001 analyzed by the ordinary one‐way ANOVA with Tukey's multiple comparison test.

We next explored the mechanisms underlying the high defense ability of 3D CRCs against ferroptosis. Previous studies uncovered that tumor cells with mesenchymal phenotype are more sensitive to ferroptosis inducer, suggesting the association between EMT state and ferroptosis.^[^ ^18^, ^35^ ^]^ We then characterized the EMT phenotype of 2D and 3D CRCs. To our surprise, 3D CRCs exhibited much lower expression of E‐cadherin than 2D cells (Figure S2A, Supporting Information), as it is known to enhance the resistance of tumor cells to ferroptosis.^[^ ^18^ ^]^ Further, 3D CRCs expressed both epithelial (keratin 5/14/19: *KRT5*, *KRT14*, *KRT19*) and mesenchymal (*ZEB1*, *ZEB2*, *VIMENTIN*) markers (**Figure** **2**A–C), especially *KRT19* and *ZEB2*. The coexpressions of epithelial and mesenchymal markers were further confirmed in individual 3D CRCs (Figure 2D). We further performed flow cytometry analysis using specific cell surface molecules CD44 and CD104 (integrin β4, ITGB4),^[^ ^8^ ^]^ which are typical markers of mesenchymal and epithelial phenotype in CRCs,^[^ ^36^, ^37^, ^38^, ^39^, ^40^ ^]^ respectively. The results showed that the majority of 2D cells (90.3% and 89.9%) showed a mesenchymal phenotype with low expression of CD104 and high expression of CD44, whereas the majority of 3D CRCs (91.2% and 89.2%) were CD44^+^CD104^+^, representing a typical hybrid EMT phenotype^[^ ^8^ ^]^ (Figure 2E,F; Figure S2B, Supporting Information). The hybrid EMT state is also characterized by the activation of canonical WNT pathway.^[^ ^8^ ^]^ 3D CRCs exhibited decreased phosphorylated β‐catenin recognized by GSK3β, elevated canonical *WNT7A/B*, *LRP6*, and *TCF4*, and nuclear localization of β‐catenin (Figure 2G–I; Figure S2C, Supporting Information), suggesting the activation of WNT/β‐catenin signaling. FAT deficiency has been reported to induce a hybrid EMT state in squamous cell carcinomas.^[^ ^41^ ^]^ FAT1 deletion did greatly elevate the proportion of CD44^+^CD104^+^ subpopulation and ferroptosis resistance of 2D CRCs (Figure S2D–G, Supporting Information). These findings demonstrate that 3D CRCs assume a hybrid EMT state, implicating the association between hybrid EMT and ferroptosis defense.

To investigate the mechanisms underlying the hybrid EMT of 3D CRCs, we assessed the roles of matrix stiffness, dimensionality, and composition or ligand. We first performed a time‐course experiment where 3D cells were transferred onto 2D rigid dishes for 1 to 3 days. After just 1 day, the mRNA levels of both epithelial and mesenchymal markers returned to similar levels of 2D cells (Figure S3A, Supporting Information), indicating that the EMT state of cells is sensitive to physical environment. Next, we examined the impact of substrate stiffness on EMT plasticity by culturing cells on 2D polyacrylamide (PA) gels with varying rigidity and coated with either fibrinogen or collagen I. The expressions of mesenchymal markers were significantly reduced in a rigidity‐dependent manner (Figure S3B, Supporting Information). However, the rigidity of 2D substrates did not effectively promote hybrid EMT (Figure 2J,K, Supporting Information). In contrast, CRCs cultured in 3D collagen gels increased both *KRT19* and *ZEB2*, and contained ≈40–56% of CD44^+^CD104^+^ subpopulation (Figure 2J,K; Figure S3C, Supporting Information). We further investigated the impact of matrix softness‐dependent stemness markers^[^ ^29^ ^]^ on hybrid EMT phenotype. Overexpression of stemness markers was not sufficient to drive hybrid EMT, but rather dramatically upregulated *ZEB2* expression and decreased cellular resistance to ferroptosis (Figure S3D–G, Supporting Information). Matrix stiffness‐mediated stemness may sustain the mesenchymal phenotype of 3D CRCs.

**Figure 3:**
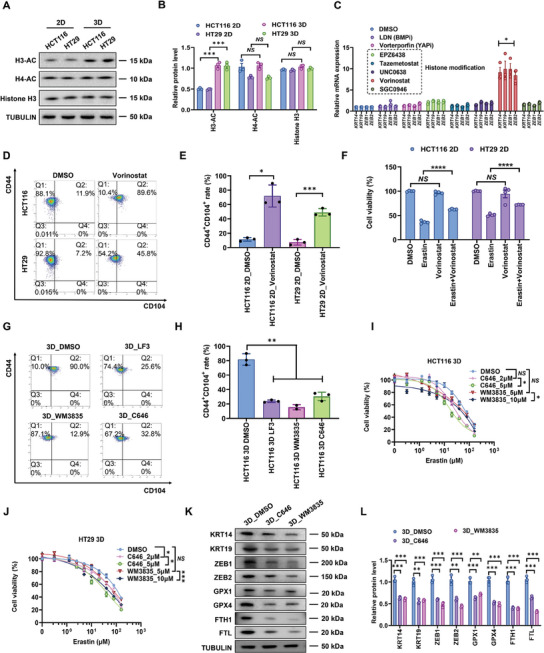
Histone acetylation promotes ferroptosis resistance of 3D CRCs. A,B) H3‐AC, H4‐AC, Histone H3, and TUBULIN protein levels in 2D and 3D CRCs were detected by western blotting. Relative protein levels of each protein were normalized to TUBULIN. Means ± SEMs; *n* = 3. NS, nonsignificant difference, ***P < 0.001 analyzed by two‐tailed Student's *t*‐test. C) 2D HCT116 cells were treated with DMSO, or inhibitors of BMP, YAP, and histone modifications. Total mRNA was extracted to detect the expression of epithelial and mesenchymal markers. Means ± SEMs; *n* = 3. *P < 0.05 analyzed by the ordinary one‐way ANOVA with Tukey's multiple comparison test. D,E) 2D CRCs were treated with HDAC inhibitor Vorinostat (1 µm) for 24 h. Then flow cytometry profiles for CD104/CD44 were detected. Means ± SEMs; *n* = 3. *P < 0.05, ***P < 0.001 analyzed by two‐tailed Student's *t*‐test. F) 2D CRCs were treated with DMSO, 1 µm Vorinostat and 10 µm Erastin accordingly. After 24 h, relative cell viability of cells was detected. Means ± SEMs; *n* = 4. NS, nonsignificant difference, ****P < 0.0001 analyzed by the ordinary one‐way ANOVA with Tukey's multiple comparison test. G,H) Flow cytometry profiles for CD44/CD104 of 3D HCT116 cells treated with DMSO, 5 µm LF3 (an antagonist of β‐catenin/TCF4 interaction), 5 µm C646 or 10 µm WM3835 for 24 h. Means ± SEMs; *n* = 3. **P < 0.01 analyzed by the ordinary one‐way ANOVA with Tukey's multiple comparison test. I,J) Relative cell viability of 3D CRCs treated with DMSO or different concentrations of Erastin for 24 h after pretreatment by histone acetylation inhibitors, C646 (2, 5 µm) or WM3835 (5, 10 µm) for 12 h. Means ± SEMs; *n* = 4. *P < 0.05, ***P < 0.001 analyzed by the ordinary one‐way ANOVA with Tukey's multiple comparison test. K,L) 3D cultured HCT116 cells were treated with DMSO, or histone acetylation inhibitors, C646 (5 µm) or WM3835 (10 µm) for 24 h. KRT14, KRT19, ZEB1, ZEB2, GPX1, GPX4, FTH1, FTL, and TUBULIN protein levels were detected by western blotting. Relative protein levels of each protein were normalized to TUBULIN. Means ± SEMs; *n* = 4. NS, nonsignificant difference, **P < 0.01, ***P < 0.001 analyzed by the ordinary one‐way ANOVA with Tukey's multiple comparison test.

Our previous studies show the prominent effects of 3D soft fibrin on BMP, YAP, and histone modifications in CRCs.^[^ ^25^, ^29^, ^42^ ^]^ Indeed, 3D CRCs exhibited higher levels of histone H3 acetylation (H3‐AC) (**Figure** **3**A,B). The inhibition of histone deacetylation but not BMP nor YAP in 2D CRCs promoted the expressions of epithelial and mesenchymal markers and increased the percentage of CD44^+^CD104^+^ subpopulation, whereas inhibition of histone acetylation in 3D CRCs suppressed hybrid EMT phenotype (Figure 3C–L). Consistent with our findings, histone deacetylases (HDACs) have been reported to inhibit EMT in CRC.^[^ ^43^, ^44^ ^]^ Histone acetylation activates WNT/β‐catenin signaling in B‐cell acute lymphoblastic leukemia cells, implying a potential link between histone acetylation and hybrid EMT.^[^ ^45^ ^]^ Therefore, these results suggest that histone acetylation modification may be involved in maintaining the hybrid EMT state of 3D CRCs.

In summary, our results conclude that 3D fibrin‐mediated histone acetylation likely induces the hybrid EMT phenotype of 3D‐cultured CRCs.

### 2.3 Histone Acetylation‐Mediated WNT/β‐Catenin Signaling Promotes Hybrid EMT‐Dependent Resistance of 3D CRCs to Ferroptosis

**Figure 4:**
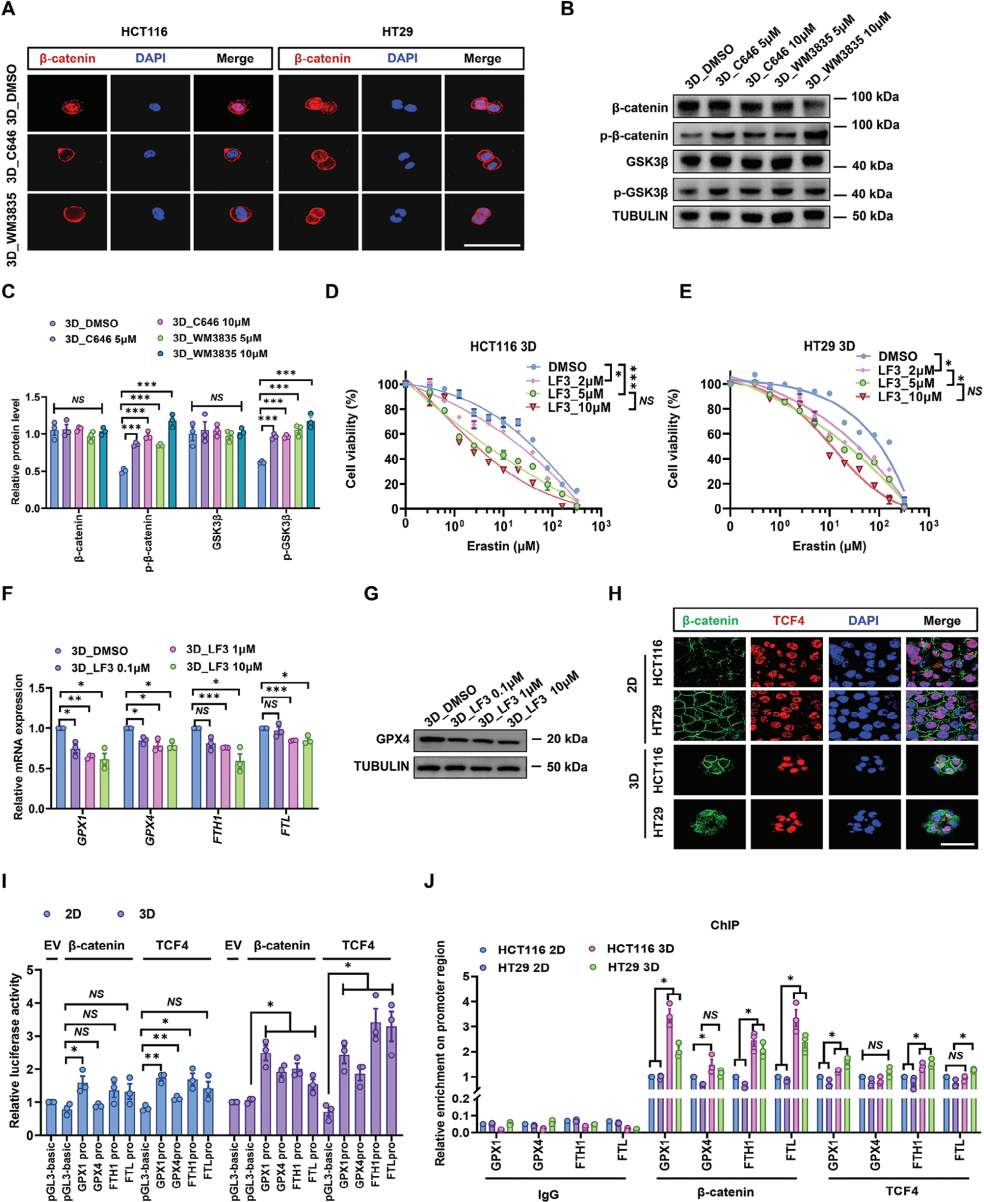
Histone acetylation‐mediated WNT/β‐catenin signaling promotes hybrid EMT‐dependent resistance of 3D CRCs to ferroptosis. A) CRCs were cultured in 3D for 2 h and then treated with 5 µm C646 or 10 µm WM3835 for 10 h. Cells were stained by β‐catenin (red) showing differential subcellular localization. Nuclei were visualized by DAPI (blue). Scale bar: 50 µm. At least three colonies were randomly selected for each condition. B,C) 3D cultured HCT116 cells were treated with DMSO, C646 (2, 5 µm) or WM3835 (5 and 10 µm) for 24 h. Phospho‐β‐catenin, total β‐catenin, phospho‐GSK3β, total GSK3β, and TUBULIN protein levels were detected by western blotting. Relative protein levels of each protein were normalized to TUBULIN. Means ± SEMs; *n* = 3. NS, nonsignificant difference, ***P < 0.001 analyzed by the ordinary one‐way ANOVA with Tukey's multiple comparison test. D,E) Relative cell viability of 3D CRCs treated with DMSO or different concentrations of Erastin for 24 h after pretreatment by different concentrations of LF3 (2, 5, 10 µm) for 12 h. Means ± SEMs; *n* = 4. NS, nonsignificant difference, *P < 0.05, ***P < 0.001 analyzed by the ordinary one‐way ANOVA with Tukey's multiple comparison test. F,G) 3D cells were treated with different concentrations of LF3 (0.1, 1, and 10 µm) for 24 h. Total mRNA and protein were extracted to detect the expression of GPX1, GPX4, FTH1, and FTL. Means ± SEMs; *n* = 3. NS, nonsignificant difference, *P < 0.05, **P < 0.01, ***P < 0.001 analyzed by the ordinary one‐way ANOVA with Tukey's multiple comparison test. H) Cells were fixed and stained by β‐catenin (green) and TCF4 (red). Nuclei were visualized by DAPI (blue). Scale bar: 50 µm. At least three views were randomly selected for each group. I) The interaction between β‐catenin/TCF4 protein and indicate genes was verified respectively in 2D and 3D CRCs by dual luciferase reporter assay. Results are given as the ratio of firefly to renilla luciferase (internal control) and were normalized to a promoter‐less construct (pGL3‐basic). Means ± SEMs; *n* = 3. NS, nonsignificant difference, *P < 0.05, **P < 0.01 analyzed by the ordinary one‐way ANOVA with Tukey's multiple comparison test. J) The interaction between β‐catenin/TCF4 protein and indicated genes was verified respectively in 2D and 3D CRCs by ChIP assay. The enrichment of β‐catenin/TCF4 protein on the indicated gene promoter was analyzed by qPCR. Means ± SEMs; *n* = 3. NS, nonsignificant difference among all indicated groups, *P < 0.05 analyzed by two‐tailed Student's *t*‐test.

Our results have shown that 3D‐cultured CRCs exhibit ferroptosis defense and histone acetylation‐mediated hybrid EMT phenotype. HDACs have been recognized as regulators of ferroptosis and redox homeostasis.^[^ ^46^, ^47^ ^]^ These findings suggest the potential roles of hybrid EMT in ferroptosis resistance. To test this idea, the hybrid EMT state was modulated by targeting histone acetylation and WNT/β‐catenin signaling. Pharmacologic inhibition of HDACs in 2D control cells promoted the hybrid EMT phenotype and significantly enhanced the resistance to FIN‐induced ferroptosis (Figure 3D–F). On the other hand, the inhibition of histone acetylation in 3D‐cultured CRCs reduced the expressions of both epithelial and mesenchymal markers and decreased the percentage of CD44^+^CD104^+^ subpopulation from ≈90% to 13–33% (Figure 3G,H), suggesting the loss of the hybrid EMT phenotype. Notably, inhibiting histone acetylation significantly decreased the level of ferroptosis defense (Figure 3I,J). This was possibly due to the suppressive effects of histone acetylation inhibition on WNT/β‐catenin signaling and the proteins related to glutathione metabolism (GPX1 and GPX4) and iron metabolism (FTH1 and FTL) (Figure 3K,L; **Figure** **4**A,C).

Since WNT/β‐catenin signaling is critical in maintaining the hybrid EMT state,^[^ ^8^ ^]^ we further examined its effect on the resistance to ferroptosis. The inhibition of this pathway with LF3, an inhibitor of the WNT/β‐catenin transcriptional domain TCF4, reduced the fraction of subpopulation with hybrid EMT phenotype in 3D CRCs from ≈90% to ≈25% (Figure 3F,G). Remarkably, inhibition of TCF4 resulted in a dose‐dependent loss of ferroptosis resistance in 3D CRCs (Figure 4D,E; Figure S4A, Supporting Information). Conversely, the treatment of 2D CRCs with recombinant human WNT3A protein, a canonical WNT inducer, or BIO, a GSK‐3 inhibitor that activates WNT signaling, improved the defense against ferroptosis (Figure S4B,C, Supporting Information). β‐catenin transcriptionally regulates GPX4, a critical enzyme involved in ferroptosis regulation.^[^ ^48^ ^]^ We further examined the influence of WNT/β‐catenin signaling on the key regulators of ferroptosis. LF3 or BIO resulted in dose‐dependent changes in both mRNA and protein levels of these genes (Figure 4F,G; Figure S4D,E, Supporting Information). To explore the mechanism underlying this regulation, we performed a global analysis of the known ferroptosis regulators in our RNA‐seq results, including GSH/GPX4, ferroptosis suppressor protein (FSP1)/CoQ10, dihydroorotic acid dehydrogenase (DHODH)/CoQH2, GTP cyclohydrolase 1 (GCH1)/tetrahydrobiopterin (BH4), lipid metabolism, and iron metabolism pathways.^[^ ^14^ ^]^ GSH/GPX4 and iron metabolism pathways but not others were significantly altered in 3D compared to 2D CRCs (Figure S5A,B, Supporting Information). It appeared that those conserved pathways were not affected by the modulation of histone acetylation and WNT/β‐catenin activity (Figure S5C–F, Supporting Information). Therefore, we next investigated the interaction between β‐catenin/TCF4 and GPXs/ferritin, showing the colocalization of β‐catenin with TCF4 in 3D CRCs (Figure 4H). The dual luciferase reporter gene assay and chromatin immunoprecipitation (ChIP) analysis (Figure 4I,J) clarified the enrichment of β‐catenin/TCF4 in *GPX1* and *FTH1* promoter regions of 3D CRCs. These findings implicate that nuclear β‐catenin in 3D CRCs sustains their hybrid EMT phenotype and transcriptionally upregulates the expressions of *GPX1* and *FTH1*, which in turn enhance their defense against ferroptosis.

### 2.4 E‐Cadherin Deficiency in 3D CRCs Mediates a CD61 Labeled‐Late Hybrid EMT State and Promotes the Superdefense against Ferroptosis

**Figure 5:**
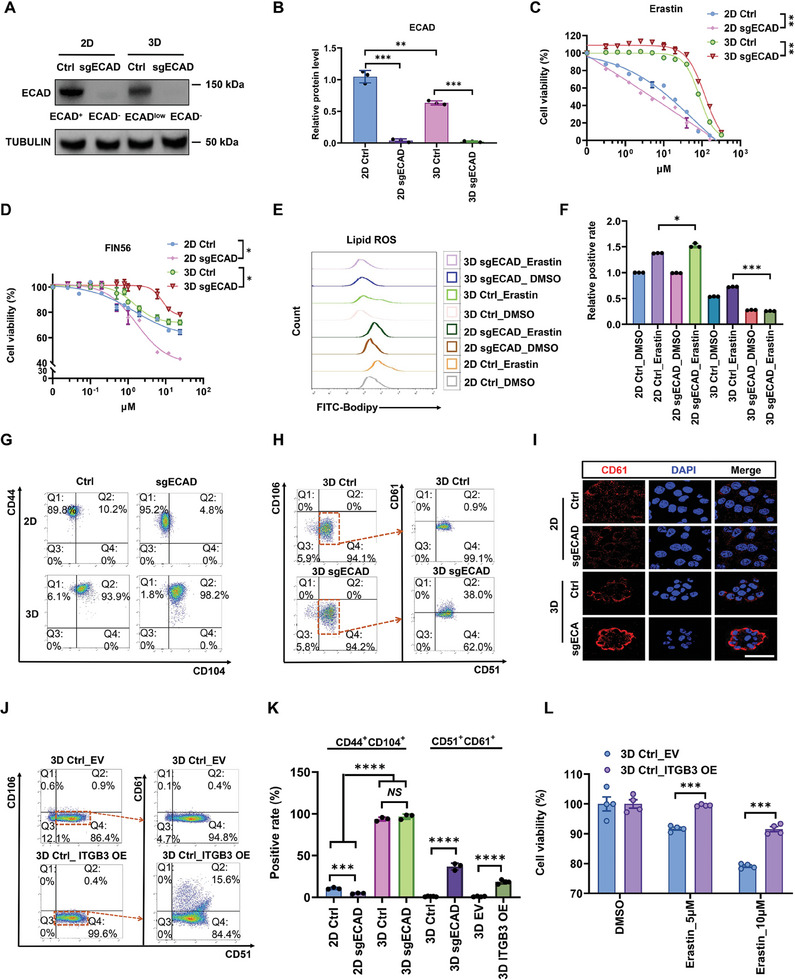
E‐cadherin deficiency in 3D CRCs mediates a late hybrid EMT state and promotes the superdefense against ferroptosis. A,B) HT29 cells were transfected with nontargeting control (Ctrl), or sgRNA targeting E‐cadherin (sgECAD), and then cultured on rigid plastic or in 90‐Pa 3D fibrin gels for 3 days. TUBULIN and ECAD protein expression levels were determined by western blotting. *n* = 3. **P < 0.01, *** *P* < 0.001 analyzed by the ordinary one‐way ANOVA with Tukey's multiple comparison test. C,D) Relative cell viability of 2D Ctrl, 2D sgECAD, 3D Ctrl, and 3D sgECAD cells treated with different concentrations of Erastin or FIN56 for 24 h. Means ± SEMs; *n* = 4. *P < 0.05, **P < 0.01 analyzed by the ordinary one‐way ANOVA with Tukey's multiple comparison test. E,F) 2D Ctrl, 2D sgECAD, 3D Ctrl, and 3D sgECAD cells were treated with DMSO or 10 µm Erastin for 24 h. Lipid ROS was measured using a BODIPY‐C11 probe by flow cytometry. Means ± SEMs; *n* = 4. *P < 0.05, **P < 0.01, ***P < 0.001 analyzed by the ordinary one‐way ANOVA with Tukey's multiple comparison test. G) Flow cytometry profiles for CD104 and CD44 of 2D Ctrl, 2D sgECAD, 3D Ctrl, and 3D sgECAD cells. *n* = 3. H) Flow cytometry profiles for CD106/CD51/CD61 of 3D Ctrl, and 3D sgECAD cells. *n* = 3. Cells in orange dashed boxes (CD106^−^/CD51^+^) were further assayed by CD61 antibody. I) Cells were fixed and stained with CD61 antibody (red) and DAPI (blue). Scale bar: 50 µm. At least three views were randomly selected for each group. J) HT29 cells were transfected with empty vector (EV), or ITGB3 overexpression plasmid (OE), then cultured in 3D. Flow cytometry profiles for CD106/CD51/CD61 of 3D Ctrl_EV and 3D Ctrl_ ITGB3 OE cells. *n* = 3. K) Quantitative analysis of the CD44/CD104 and CD51/CDD61 positive rate for (G), (H), and (J). Means ± SEMs; *n* = 3. NS, nonsignificant difference, ***P < 0.001, ****P < 0.0001 analyzed by the ordinary one‐way ANOVA with Tukey's multiple comparison test. L) Relative cell viability of 3D Ctrl_EV and 3D Ctrl_ITGB3 OE treated with 5 or 10 µm Erastin for 24 h was detected. Means ± SEMs; *n* = 4. ***P < 0.001 analyzed by two‐tailed Student's *t‐*test.

Our results indicated an unexpected association between low expression of E‐cadherin and high defense against ferroptosis in 3D‐cultured CRCs (Figure S2A, Supporting Information), which seems to be different from previous findings that E‐cadherin can promote the resistance of 2D cells to ferroptosis. It has been reported that E‐cadherin‐mediated intercellular interactions inhibit ferroptosis via NF2‐YAP‐ACSL4 axis.^[^ ^18^ ^]^ To investigate whether E‐cadherin played distinct roles in the ferroptosis defense of 2D and 3D CRCs, E‐cadherin was knocked out (sgECAD) using the CRISPR‐Cas9 technique (**Figure** **5**A,B). The clones with unaffected cell proliferation after E‐cadherin deletion in 3D were chosen for subsequent experiments (Figure S6A–C, Supporting Information). It was observed that 2D cells lacking E‐cadherin showed increased sensitivity to ferroptosis (Figure 5C–F; Figure S6D, Supporting Information), which is consistent with previous reports.^[^ ^18^ ^]^ Surprisingly, E‐cadherin‐deleted 3D cells (ECAD^−/−^) were even more resistant to ferroptosis compared to 3D control cells (ECAD^low^, about half of 2D cells) when exposed to FINs such as Erastin or FIN56 (Figure 5A–F; Figure S6D–F, Supporting Information). Interestingly, E‐cadherin deletion did not considerably affect the proportion of CD44^+^CD104^+^ subpopulation and activation level of WNT/β‐catenin in 3D CRCs (Figure 5G; Figure S7A,B, Supporting Information). Therefore, we further examined whether 3D CRCs with and without E‐cadherin deletion were two subpopulations with distinct hybrid EMT phenotype. Both 3D Ctrl and sgECAD CRCs were negative for CD106 (also known as vascular cell adhesion molecule VCAM1), while 3D Ctrl CRCs expressed CD51 (integrin α_v_ or ITGAV) alone and ≈38% of 3D sgECAD cells were CD51^+^CD61^+^ (Figure 5H,I), suggesting that 3D CRCs with E‐cadherin deficiency are at the late stage of hybrid EMT (Figure S7C, Supporting Information).^[^ ^7^ ^]^ Intriguingly, overexpression of CD61 (integrin β_3_ or ITGB3) in 3D cells increased the proportions of CD51^+^CD61^+^ cells from less than 1% to ≈15%, and enhanced their resistance to ferroptosis, implying that the unique expression of CD61 in 3D sgECAD cells with late‐hybrid EMT phenotype might account for their enhanced resistance to ferroptosis (Figure 5J–L; Figure S7D, Supporting Information). These results conclude that E‐cadherin deletion induces a late stage of hybrid EMT phenotype and superdefense against ferroptosis in 3D‐cultured CRCs.

### 2.5 Cell–ECM Adhesion‐Mediated Tension Counteracts Ferroptosis via Mitochondrial Reprogramming

To understand how E‐cadherin‐deficient 3D cells acquire enhanced ferroptosis defense, we investigated the expressions of EMT‐associated genes as well as known ferroptosis regulators (e.g., GPX1, GPX4, FTH1, FTL, ACSL4, FSP1, SLC7A11, and DHODH) in 3D Ctrl and 3DsgECAD CRCs, which showed no obvious changes (Figure S8A–C, Supporting Information). We further examined the effects of EMT transcription factors on ferroptosis. Ectopic expression of *ZEB1* significantly reduced the ferroptosis defense of 2D but not 3D CRCs, while silencing *ZEB1* or *ZEB2* promoted the antiferroptosis ability of both 2D and 3D tumor cells (Figure S8D–F, Supporting Information). Furthermore, the inhibition of HDACs increased the ferroptosis defense in both 2D Ctrl and 2D sgECAD CRCs but did not render sgECAD cells more resistant to ferroptosis than 2D Ctrl (Figure S8G, Supporting Information). These findings suggest that other unknown mechanisms may mediate the superdefense against ferroptosis of E‐cadherin‐deficient 3D CRCs.

**Figure 6:**
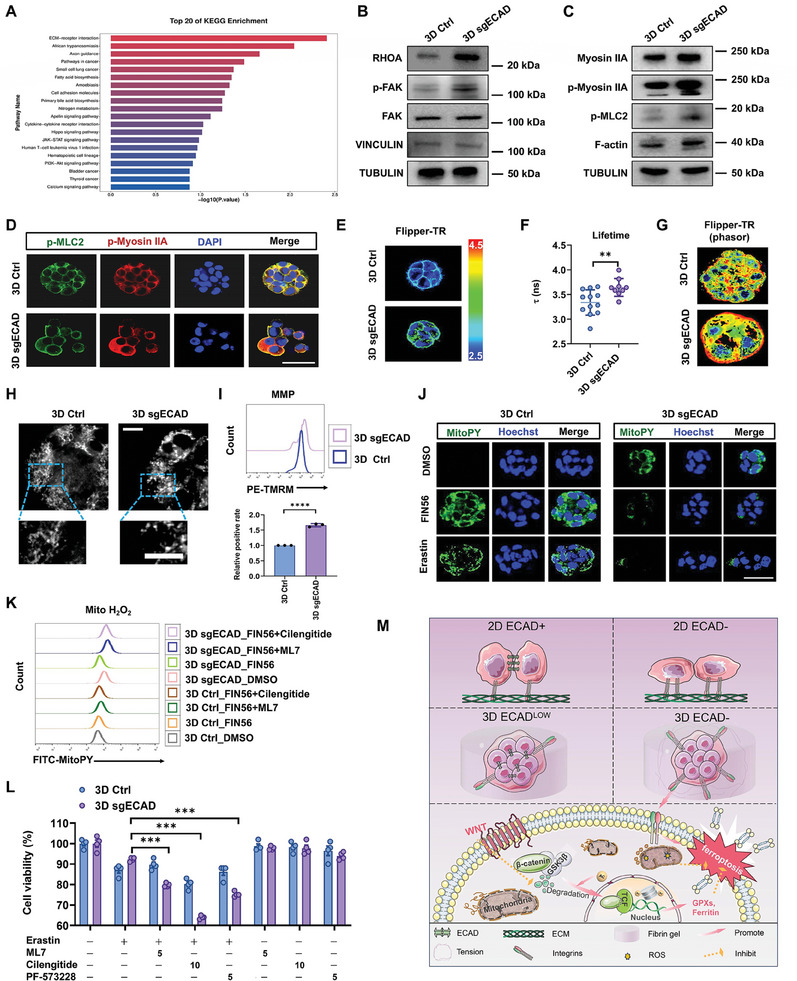
Cell–ECM adhesion‐mediated tension counteracts ferroptosis via mitochondrial reprogramming. A) KEGG pathway analysis of the pathways enriched by differentially expressed genes in 3D Ctrl and 3D sgECAD cells (retaining the top 20). Means ± SEMs; *n* = 3. B) RHOA, FAK, phospho‐FAK, VINCULIN, and TUBULIN protein levels in 3D Ctrl, and 3D sgECAD cells cultured for 3 days were detected by western blotting. *n* = 3. C) Myosin IIA, phospho‐MyosinIIA, phospho‐MLC2, F‐actin, and TUBULIN protein levels in 3D Ctrl and 3D sgECAD cells cultured for 3 days were detected by western blotting. *n* = 3. D) 3D Ctrl and 3D sgECAD cells were fixed and stained by phospho‐MLC2 (green) and phospho‐MyosinIIA (red). Nuclei were visualized by DAPI (blue). Scale bar: 50 µm. At least three colonies were randomly selected for each group. E) Fast FLIM images of 3D Ctrl and 3D sgECAD colonies pseudocolored according to their membrane tension labeled by Flipper‐TR probe. FLIM images were acquired by Leica Sp8 FLIM microscope. F) Quantitative fluorescence lifetime analysis of cell membrane tension. Means ± SEMs; *n* = 12, and 9, respectively. **P < 0.01 analyzed by two‐tailed Student's *t*‐test. G) Phasor FLIM analysis was performed to visualize the distributions of membrane tension fluorescent lifetime. Colors were indicative of the frequency of photons (red = high, blue = low). H) Confocal microscopy depicting mitochondrial network structure in 3D Ctrl and 3D sgECAD cells stained with 200 nm mitotracker for 30 min. Scale bar: 10 µm. At least three views were randomly selected for each group. I) The mitochondrial membrane potential (MMP) of 3D Ctrl and 3D sgECAD cells was measured with flow cytometry after being stained with 20 nm Tetramethylrhodamine (TMRM) for 30 min. Means ± SEMs; *n* = 3. ****P < 0.0001 analyzed by two‐tailed Student's *t*‐test. J) 3D Ctrl, and 3D sgECAD cells were treated with DMSO, 3 µm FIN56, 10 µm Erastin for 24 h. The mitochondrial hydrogen peroxide level of cells was imaged with confocal microscopy after being stained with 5 µm mitoPY‐1 for 1 h. Scale bar: 40 µm. At least three colonies were randomly selected for each condition. K) 3D Ctrl and 3D sgECAD cells were pretreated with DMSO, 10 µm ML7, and 10 µm Cilengitide for 6 h and then treated with DMSO or 3 µm FIN56 for 24 h. Mitochondrial hydrogen peroxide (Mito H_2_O_2_) level of 3D Ctrl and 3D sgECAD cells was measured with flow cytometry after being stained with 5 µm mitoPY‐1 for 1 h. *n* = 3. L) 3D Ctrl and 3D sgECAD cells were pretreated with DMSO, 10 µm Cilengitide for 6 h, 5 µm ML7, or FAK Inhibitor PF‐573228 (5 µm) for 24 h and then treated with DMSO or 15 µm Erastin for 24 h. Relative cell viability of cells was detected. Means ± SEMs; *n* = 4. ***P < 0.001 analyzed by the ordinary one‐way ANOVA with Tukey's multiple comparison test. M) A schematic diagram illustrated the distinct contributions of EMT states (2D or 3D; with or without E‐cadherin) to CRC ferroptosis involving histone acetylation–WNT/β‐catenin–GPXs/ferritin signaling and adhesion tension‐mediated mitochondrial reprogramming.

Through transcriptomic analysis, we identified prominent differences in ECM–receptor interaction and focal adhesion pathways between 3D Ctrl and sgECAD cells (**Figure** **6**A; Figure S9A, Supporting Information). Knockout of E‐cadherin activated the proteins associated with cell–ECM adhesion, especially actomyosin‐mediated cell tension and focal adhesion, including the upregulation of RHOA, phosphorylation of Myosin IIA and MLC2, F‐actin, and phosphorylation of FAK (Figure 6B–D; Figure S9B,C, Supporting Information). These findings suggest the upregulation of cellular tension after E‐cadherin deletion and were reminiscent of the effects of CD61 overexpression on 3D CRCs (Figures S7D and S9D, Supporting Information). To test this possibility, we utilized Flipper‐TR, a living cell fluorescent membrane tension probe combined with fluorescence lifetime imaging microscopy (FLIM), to evaluate cell membrane tension. The results showed a significant increase of membrane tension in 3D sgECAD cells compared to 3D Ctrl cells (Figure 6E,F). Further phasor FLIM analysis revealed that high membrane tension observed in 3D sgECAD cells was mainly concentrated in the periphery of the colonies, where 3D cells interacted with the surrounding matrices through integrin‐mediated adhesion (Figure 6G), suggesting the influence of E‐cadherin deficiency on the spatial distribution of cell membrane tension throughout living cells.

We identified the late hybrid EMT phenotype of 3D sgECAD cells (Figure 5H,I; Figure S8C, Supporting Information), characterized by the expressions of CD51 (integrin α_v_) and CD61 (integrin β_3_). CRCs in 3D fibrin gels interacted with matrix ligands through integrins α_v_β_3_,^[^ ^25^, ^49^ ^]^ suggesting that these integrins might be responsible for the enhanced cell‐matrix adhesion and cell membrane tension. It has been reported that adhesion‐mediated tension can influence mitochondrial structure and function, leading to mitohormesis and reshaping of oxidative stress resilience (OxSR).^[^ ^50^ ^]^ Notably, both Erastin and FIN56 could induce mitochondrial ROS production (Figure S10A, Supporting Information). We thus hypothesized that the increase of adhesion‐mediated tension in 3D sgECAD cells might play a role in their defense against ferroptosis by remodeling the OxSR of mitochondria. The results showed that 3D sgECAD cells exhibited fragmented/toroidal mitochondrial morphology (Figure 6H) but maintained a high mitochondrial membrane potential (Figure 6I; Figure S10B, Supporting Information), typical features of mechanosignaling‐induced mitohormesis.^[^ ^50^ ^]^ In comparison, 3D Ctrl cells had mitochondria with thin interconnected filaments (Figure 6H). Furthermore, 3D sgECAD cells exhibited higher basal levels of mitochondrial hydrogen peroxide under DMSO treatment and higher ROS scavenging upon exposure to Erastin or FIN56 compared to 3D Ctrl cells (Figure 6J,K). Importantly, the inhibition of myosin light chain kinase by ML7, focal adhesion kinase by PF‐573228, or integrins α_ν_β_3_ by Cilengitide^[^ ^51^ ^]^ interfered with adhesion‐mediated tension, and suppressed the mitohormesis and the high resistance of 3D sgECAD cells to ferroptosis (Figure 6K,L; Figure S10C,D, Supporting Information). In contrast, similar treatments did not affect the susceptibility of 2D cells to ferroptosis (Figure S10E–G, Supporting Information). These findings demonstrate that integrin‐mediated focal adhesion and cellular tension in 3D sgECAD cells with late hybrid EMT state induce mitochondrial reprogramming and further contribute to their superior tolerance to ferroptosis.

To clarify the roles of E‐cadherin in driving late hybrid EMT and ferroptosis susceptibility, we examined the effects of E‐cadherin knockdown or overexpression on 3D Ctrl cells by lentiviral infection (Figure S11, Supporting Information). Surprisingly, although knockdown of E‐cadherin upregulated the proportion of CD61, it had little effect on the sensitivity of 3D cells to ferroptosis. On the other hand, E‐cadherin overexpression had subtle effects on the ferroptosis of 3D CRCs, which might be due to the downregulation of Acyl‐CoA synthetase long‐chain family 4 (ACSL4), a known downstream effector of E‐cadherin expression^[^ ^18^ ^]^ and promotor for ferroptosis^[^ ^52^ ^]^ (Figure S12, Supporting Information).

To conclude, different EMT phenotypes play distinct roles in the ferroptosis of 2D and 3D CRCs: E‐cadherin‐deficient 2D CRCs with mesenchymal state are more sensitive to ferroptosis than 2D control cells; 3D CRCs with E‐cadherin exhibit hybrid EMT phenotype and high level of resistance to ferroptosis via histone acetylation–WNT/β‐catenin–GPXs/ferritin signaling axis, while E‐cadherin deletion further enhances the resistance of 3D cells with CD61 (integrin β_3_) marked‐late hybrid EMT state to ferroptosis through integrin‐mediated tensioning and mitochondrial reprogramming (Figure 6M).

### 2.6 The Inhibition of WNT/β‐Catenin Signaling and Integrin α_v_β_3_ Sensitizes 3D CRCs to Ferroptosis In Vivo

**Figure 7:**
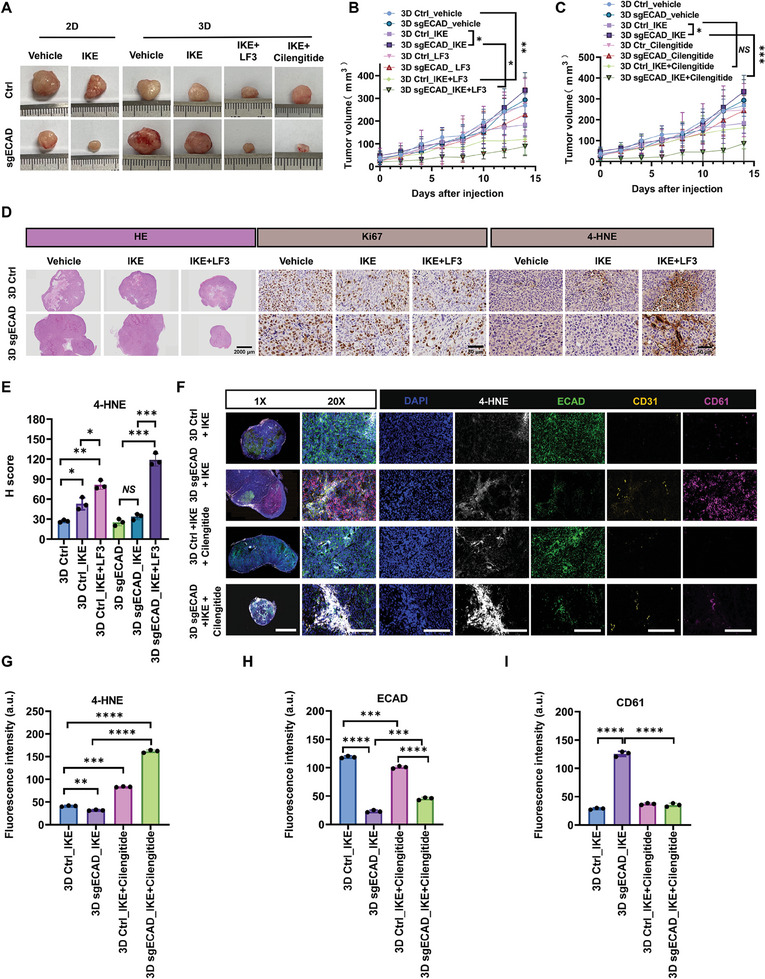
The inhibition of WNT/β‐catenin signaling and integrin α_v_β_3_ sensitizes 3D CRCs to ferroptosis in vivo. A) Representative images of tumor tissue from different mice. Cells were subcutaneously injected into BALB/c nude mice after adjusting the cell concentration to 10^6^. Once the tumors reached a diameter of ≈5 mm, mice were treated with vehicle, IKE (50 mg kg^−1^), IKE + LF3 (50 mg kg^−1^), or IKE + Cilengitide (100 µg) by intraperitoneal injection every two days for 14 days. At least six mice for each group. B,C) Statistical analysis of the tumor volumes for 14 days. At least six mice for each group. Means ± SEMs; NS, nonsignificant difference, *P < 0.05, **P < 0.01, ***P < 0.001 analyzed by the ordinary two‐way ANOVA with Tukey's multiple comparison test. D) Hematoxylin and eosin (HE) (scale bar: 2000 µm), Ki‐67 and 4‐HNE staining (scale bar: 50 µm) of 3D Ctrl and 3D sgECAD tumor tissues of mice treated with vehicle, IKE (50 mg kg^−1^) or IKE + LF3 (50 mg kg^−1^). *n* = 3. E) Quantitative analysis of 4‐HNE expression based on IHC results. Images were analyzed by IHC Profiler. An H‐score was calculated using the following formula: [1 × (% cells 1+) + 2 × (% cells 2+) + 3 × (% cells 3+)] × 100. Means ± SEMs; *n* = 3. NS, nonsignificant difference, *P < 0.05, **P < 0.01, ***P < 0.001 analyzed by the ordinary one‐way ANOVA with Tukey's multiple comparison test. F) Tumor tissue sections were fixed and stained with 4‐HNE (white), ECAD (green), CD31 (yellow), and CD61 (magenta) antibodies. Nuclei were visualized by DAPI (blue). Dark red arrows pointed to CD31‐positive (yellow) cells. Scale bar: 2 mm (1×) and 100 µm (20×). At least three views were randomly selected for each condition. G–I) Quantitative analysis of 4‐HNE, ECAD, and CD61 expressions based on multiplex IHC results. Images were analyzed by Indica Labs – Area Quantification FL v2.1.2 module in Halo v3.0.311.314 analysis software. Means ± SEMs; *n* = 3. **P < 0.01, ***P < 0.001, ****P < 0.0001 analyzed by the ordinary one‐way ANOVA with Tukey's multiple comparison test.

We have demonstrated that the hybrid EMT phenotype of 3D CRCs drives enhanced resistance to ferroptosis compared to 2D cells and that E‐cadherin deficiency further promotes 3D but reduces 2D cells’ defense through integrin‐mediated tension in vitro. Further in vivo results showed that compared to 2D Ctrl cells, the tumor xenografts generated by 2D sgECAD cells were more sensitive to ferroptosis induced by Imidazole ketone erastin (IKE),^[^ ^53^ ^]^ an Erastin analog for in vivo experiments (**Figure** **7**A). In contrast, both 3D Ctrl and 3D sgECAD tumors were less sensitive to IKE (Figure 7A; Figure S13A–C, Supporting Information), and E‐cadherin deficiency further promoted tumor growth under IKE treatment (Figure 7A–C), suggesting that E‐cadherin deletion in 3D CRCs enhances the resistance to ferroptosis in vivo. Moreover, coadministration of IKE with LF3 significantly suppressed the growth of tumor xenografts generated by 3D Ctrl CRCs (Figure 7A,B), and coadministration of Cilengitide remarkably diminished the ferroptosis defense advantage of 3D sgECAD tumors over 3D Ctrl tumors (Figure 7A,C) without affecting the body weight of the mice (Figure S13B, Supporting Information). These findings were further confirmed by the immunohistochemistry (IHC) staining showing that LF3 treatment increased the sensitivity of both 3D sgECAD and Ctrl tumors to IKE treatment (indicated by 4‐HNE, a lipid peroxidation marker) and suppressed cell proliferation and thus tumor growth (Figure 7D,E; Figure S13D, Supporting Information). We further examined the EMT state of the treated xenograft cells. Compared to 2D tumors, tumor cells in 3D xenografts coexpressed both epithelial and mesenchymal markers and contained elevated proportion of hybrid EMT cells in 3D tumors (Figure S13E,F, Supporting Information).

To gain further insight into the differences between 3D Ctrl and 3D sgECAD tumors after different treatments, we performed multiplex IHC (mIHC) to provide a comprehensive view of various markers simultaneously (Figure 7F). Quantitative analyses showed that E‐cadherin deficiency conferred late hybrid EMT phenotype on 3D CRCs (upregulated CD61) that enhanced the resistance to IKE‐induced ferroptosis (reduced 4‐HNE), which was abolished by selective inhibition of integrin α_v_β_3_ (Figure 7G–I). These findings further validated the influence of E‐cadherin deficiency on ferroptosis defense and the therapeutic roles of integrin α_v_β_3_ in preventing such effects, which were observed in vitro (Figure 6). Integrin α_v_β_3_ is known to be involved in vascular endothelial cell recruitment and tumor vascularization.^[^ ^54^, ^55^ ^]^ Surprisingly, the 3D sgECAD tumors exhibited more CD31‐labeled vascular endothelial cells, which disappeared after Cilengitide coadministration (Figure 7F). This intriguing finding suggests that the late‐hybrid EMT state of tumor cells may be associated with tumor vascularization.

### 2.7 EMT State and Cell Membrane Tension Predict Therapeutic Resistance of CRC

**Figure 8:**
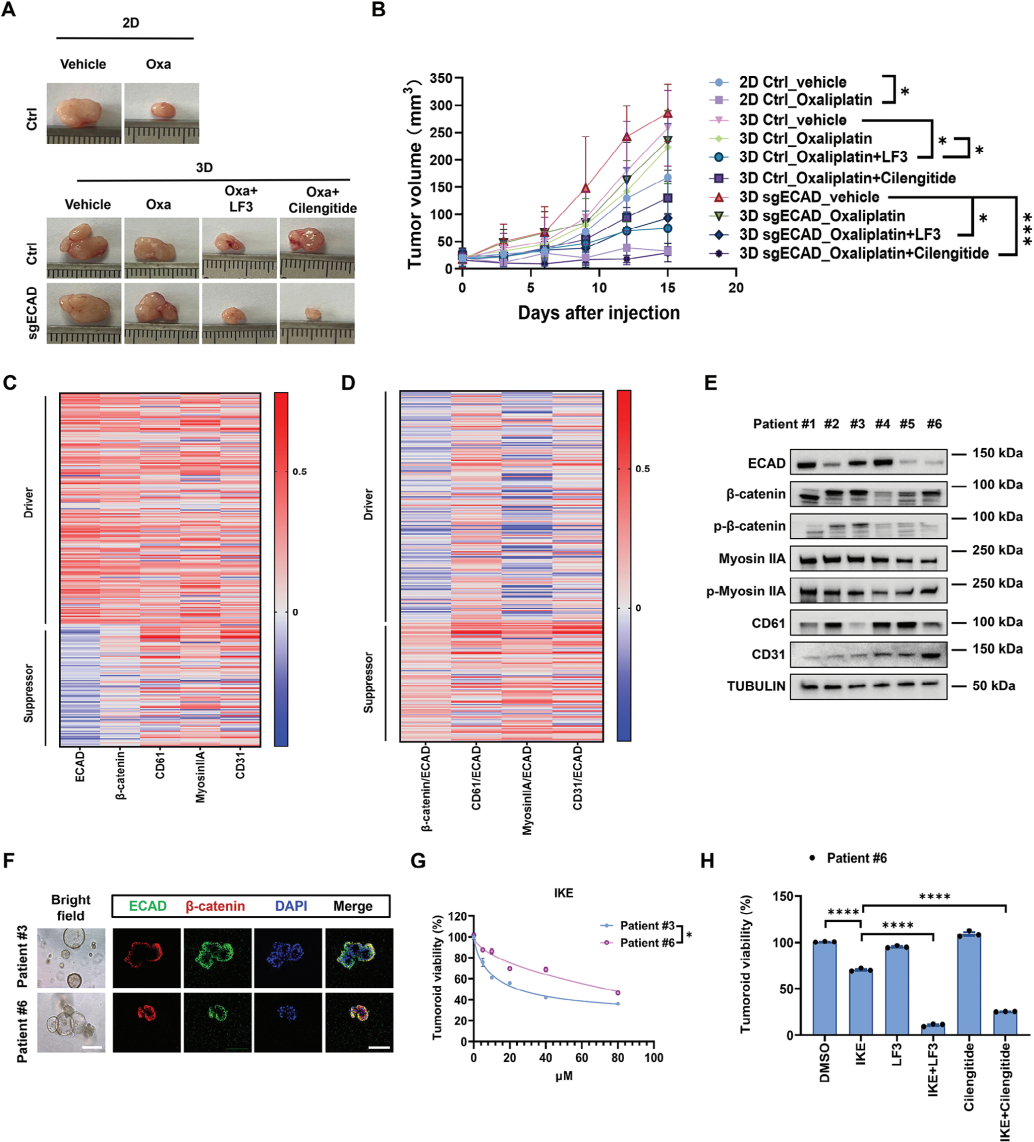
EMT states and membrane tension predict therapeutic resistance of CRC. A) Representative images of tumor tissue sections from different mice. Cells were subcutaneously injected into BALB/c nude mice after adjusting the cell concentration to 10^6^. Once the tumors reached a diameter of ≈5 mm, mice were treated with vehicle, Oxaliplatin (7.5 mg kg^−1^), Oxaliplatin+LF3 (50 mg kg^−1^), or Oxaliplatin + Cilengitide (100 µg) by intraperitoneal injection every two days for 14 days. B) Statistical analysis of the tumor volumes for 14 days. At least six mice for each group. Means ± SEMs; *P < 0.05, ***P < 0.001 analyzed by the ordinary two‐way ANOVA with Tukey's multiple comparison test. C,D) A heatmap of the correlation between the expression of hybrid EMT and membrane tension markers and ferroptosis sensitivity of CRC was provided. The expressions of positive (Driver, *n* = 183) and negative (Suppressor, *n* = 97) markers related to ferroptosis, as well as ECAD, β‐catenin, MyosinIIA, CD61, and CD31 were obtained from the TCGA COAD database. Pearson correlation analysis was used to indicate the degree of association as represented by the *r* value. Red represents a positive correlation whereas blue represents a negative correlation. The gene annotations and scores are presented in Table S5 of the Supporting Information. E) Proteins were extracted from the tumor tissue of CRC patients. ECAD, β‐catenin, phospho‐β‐catenin, MyosinIIA, phospho‐MyosinIIa, CD61, CD31, and TUBULIN protein levels were detected by western blotting. *n* = 3. F) Representative images of tumoroids from patients #3 and #6. Left: bright field images, scale bar: 500 µm. Right: IF staining for ECAD (green) and β‐catenin (red), nuclei were visualized by DAPI (blue), scale bar: 100 µm. At least three randomly selected tumoroids were captured for each group. G) Tumoroids from patients #3 and #6 were treated with DMSO or different concentrations of IKE for 24 h. Means ± SEMs; *n* = 4. *P < 0.05 analyzed by two‐tailed Student's *t*‐test. H) Tumoroids from patient #6 were pretreated with DMSO, 10 µm LF3 or 10 µm Cilengitide for 6 h, and then treated with DMSO or 10 µm IKE for 24 h. Means ± SEMs; *n* = 3. ****P < 0.0001 analyzed by the ordinary one‐way ANOVA with Tukey's multiple comparison test.

It is known that radiotherapy and Oxaliplatin, two major clinical treatments for CRC, kill tumor cells by inducing ferroptosis and oxidative stress,^[^ ^56^, ^57^ ^]^ which was recapitulated in vitro (Figure S14A,B, Supporting Information). These findings suggest that the resistance of CRC patients to the treatment‐induced ferroptosis may predict the clinical outcome. Our results have demonstrated that the hybrid EMT phenotype of 3D CRCs promotes the defense against ferroptosis, which largely depends on their antioxidant systems, including GPXs and mitohormesis. We thus examined the sensitivity of CRCs at different EMT states to these therapies. 3D and 2D sgECAD cells exhibited higher and lower levels of defense against both radiation and Oxaliplatin compared to their respective Ctrl cells, and the resistance of 3D sgECAD cells could be reversed by inhibiting adhesion tension (Figure S14C–E, Supporting Information), which were reminiscent of the responses of these cells to FIN‐induced ferroptosis. Importantly, similar findings could be reproduced in vivo when mice with xenografts generated by 2D and 3D CRCs were treated with Oxaliplatin and the corresponding coadministrations (**Figure** **8**A,B; Figure S15A, Supporting Information). To further understand the relationship between EMT state and CRC ferroptosis susceptibility, we performed a Pearson correlation analysis of hybrid EMT markers, membrane tension‐related genes, and ferroptosis regulators in the TCGA COAD database. The result indicated that E‐cadherin expression was positively correlated with the expressions of ferroptosis drivers. In addition, there were strong correlations between the ratios of β‐catenin/CD61/MyosinIIA/CD31 expression over E‐cadherin expression and ferroptosis resistance. In particular, the ratio of Myosin IIA over E‐cadherin showed the highest negative correlation with ferroptosis drivers (Figure 8C,D). These results indicate that EMT state and membrane tension of CRCs could predict their therapeutic resistance.

To establish the clinical relevance of our findings, we developed CRC patient‐derived organoids (tumoroids) for the examination of drug sensitivity. We first analyzed the molecular signatures of resected tissues from six CRC patients, in which the tumoroids from patient #3 exhibited high levels of E‐cadherin and phosphorylated β‐catenin, while patient #6 exhibited low levels of E‐cadherin and phosphorylated β‐catenin, but high levels of myosin activity, CD31, and CD61 (integrin β3), representative of CRCs with epithelial and hybrid EMT state, respectively (Figure 8E,F; Figure S15B, Supporting Information). Remarkably, the tumoroids from patient #6 showed elevated defense against ferroptosis upon exposure to IKE compared to those from patient #3 (Figure 8G). Next, we investigated whether WNT/β‐catenin pathway and adhesion tension could affect tumoroid resistance to ferroptosis. IKE treatment decreased the viability of tumoroid #6 to 70%, and cotreatment with LF3 and Cilengitide further reduced cell viability to 10% and 25% (Figure 8H), respectively, suggesting the roles of WNT/β‐catenin and integrin‐mediated tension in ferroptosis defense. Note that the treatment of LF3 or Cilengitide alone had no obvious effect on cell viability.

Taken together, these results suggest that the EMT state (represented by the levels of E‐cadherin and hybrid EMT‐associated proteins) is associated with the sensitivity of patient‐derived tumoroids to therapy‐induced ferroptosis, suggesting that the EMT phenotype of tumors may predict the therapeutic resistance to clinical treatment.

## 3 Discussion

This study reports that CRCs cultured in 3D soft fibrin microenvironment acquire hybrid EMT phenotype through the activation of canonical WNT pathway and histone acetylation, which enhances the defense against ferroptosis by transcriptionally regulating the key regulators (GPXs, ferritin) of ferroptosis. In contrast to the long‐standing notion that E‐cadherin promotes the resistance of 2D tumor cells to ferroptosis (Figure 5),^18^ deletion of E‐cadherin, a key factor in EMT, does not sensitize 3D CRCs to ferroptosis, but unexpectedly induces a late hybrid EMT state and superdefense against ferroptosis through integrin‐mediated tension and mitochondrial reprogramming. This work reveals a previously unappreciated role of hybrid EMT in ferroptosis under 3D context, which implicates the significance of intratumoral phenotypic heterogeneity and microenvironment in therapeutic resistance and the potential of targeting hybrid EMT as promising strategies in sensitizing CRCs to ferroptosis.

This work highlighted the contribution of histone acetylation–hybrid EMT‐triggered GSH/GPX4 and ion metabolism in resisting ferroptosis and proposed that mechanical force‐induced mitochondrial reprogramming is involved in ferroptosis defense, which introduces fresh perspectives for ferroptosis research. The involvement of histone modifications in disease‐associated ferroptosis is being widely discussed. Histone deacetylase 9 (HDAC9) mediates post‐translational modification of HIF‐1 and Sp1, which in turn increases TfR1 and decreases GPX4 expression, thereby promoting ferroptotic neuronal death.^[^ ^58^ ^]^ Histone demethylase KDM3B prevents ferroptosis by upregulating SLC7A11.^[^ ^59^ ^]^ Inhibition of arginine methyltransferase 1 (PRMT1) decreases the abundance of H4R3me2a in the promoter region of acyl‐CoA synthetase long‐chain family member 1 (ACSL1) and promotes ferroptosis by increasing lipid peroxidation.^[^ ^60^ ^]^ In turn, oxidative stress and iron metabolism can influence the activity of histone modification enzymes.^[^ ^61^, ^62^ ^]^ Since epigenetic alterations often lead to the development of therapeutic resistance and malignant transformation of cancer cells, a better understanding of the interplay between histone modification changes and ferroptosis may further elucidate the therapeutic potential of ferroptotic compounds. In addition, those differentially expressed genes related to the proteasome pathway (PSMB3, PSMB5, PSMB6, PSMB10) may also play a role in ferroptosis‐associated drug resistance, since many of them are involved in oxidative stress and drug resistance.^[^ ^63^, ^64^, ^65^, ^66^ ^]^ Therefore, the roles of proteasome pathways warrant further investigation in the future.

It is known that the physical microenvironment influences cellular functions mainly through the alterations of epigenetic features, which further affect the transcription of corresponding genes.^[^ ^67^, ^68^ ^]^ In line with this notion, this study shows that 3D soft fibrin increases the level of histone acetylation in CRCs. Inhibiting HDACs in 2D CRCs upregulates the expressions of both epithelial and mesenchymal markers and promotes the phenotypic conversion into hybrid EMT status while inhibiting histone acetyltransferase in 3D CRCs downregulates these proteins and abolishes the hybrid EMT phenotype. These results suggest that CRCs acquire hybrid EMT phenotype in 3D soft fibrin microenvironment through histone acetylation, which is consistent with our and other previous studies.^[^ ^20^, ^21^, ^67^ ^]^ For example, mechanical shifts at the tissue level trigger increases in nuclear size and redistribution of heterochromatin, which promote EMT phenotype and chemoresistance in invasive breast cancer.^[^ ^69^ ^]^ Previous studies indicate a correlation between EMT and ferroptosis.^[^ ^17^, ^18^, ^19^ ^]^ In particular, mesenchymal cells are more ferroptotic than epithelial counterparts. A recent study by Schwab et al. proposes that mesenchymal cell hypersensitivity to ferroptosis is dependent on ZEB1‐mediated plasma membrane remodeling (PUFA: MUFA ratio).^[^ ^70^ ^]^ Coincidentally, the mesenchymal phenotype induced by E‐cadherin deficiency or elevated ZEB1 for cellular ferroptosis was correlated with ACSL4 as well as with phospholipid composition. However, ACSL4 was not significantly altered in 3D cells (Figure S5A,B, Supporting Information), and disruption of either E‐cadherin or ZEB1 was not sufficient to increase the sensitivity of 3D cells to ferroptosis (Figure 5; Figure S8C,D, Supporting Information). These results suggest a critical role of hybrid EMT state of 3D CRCs in ferroptosis, which may not depend on ZEB1. Except for the complete epithelial and mesenchymal phenotypes, tumor cells exhibit hybrid or partial EMT state and such cells are highly malignant, supported by their stem‐like self‐renewal ability, invasion and colonization advantages, and drug resistance potential.^[^ ^71^ ^]^ However, the relationship between hybrid EMT and ferroptosis defense remains unclear. This study shows that the induction of histone acetylation and activation of WNT/β‐catenin signaling promote the hybrid EMT phenotype of 2D CRCs, which further upregulates the proteins related to ferroptosis and glutathione metabolism, enhancing the defense against ferroptosis. On the other hand, the inhibition of histone acetylation and WNT/β‐catenin signaling abolishes hybrid EMT of 3D CRCs, which sensitizes these cells to ferroptosis (Figures 3 and 4; Figure S4, Supporting Information). These findings suggest that hybrid EMT drives ferroptosis evasion, a new feature of malignant tumor cells, which provides a potential therapeutic target for the development of promising anticancer strategies.

Hybrid EMT, also known as partial EMT, is an intermediate state of EMT that coexpresses epithelial and mesenchymal markers to varying degrees.^[^ ^40^, ^72^ ^]^ Many breast and colorectal cancer cell lines have been reported to lose their epithelial phenotype through an alternative program involving protein internalization rather than transcriptional repression, resulting in a partial EMT phenotype.^[^ ^73^ ^]^ Cancer cells utilizing this procedure migrate in clusters, in contrast to the single‐cell migration pattern associated with the traditionally defined mechanism of EMT. According to Figure S7C (Supporting Information) and Pastushenko et al.,^7^ the late stage of hybrid EMT is in a more mesenchymalized hybrid state of EMT (characterized by CD51^+^CD61^+^). Despite being a quasi‐terminal stage of the EMT continuum, it is still in an intermediate state and fundamentally different from the mesenchymal state (complete EMT, characterized by CD106^+^CD51^+^CD61^+^).^[^ ^7^ ^]^ While epithelial and mesenchymal properties coexist in the hybrid EMT state, they inevitably transit dynamically across the EMT continuum.^[^ ^74^ ^]^ Despite the presence of epithelial‐associated molecules or mechanisms driven by 3D culture to maintain the epithelial properties of 3D hybrid EMT cells, E‐cadherin deficiency promotes the mesenchymalism of cells to some extent,^[^ ^7^ ^]^ and thus it is not surprising that E‐cadherin deletion drives 3D cells from comment hybrid phase (3D Ctrl CRCs, characterized by CD51^+^) to late hybrid phase that closer to a mesenchymal state.

An unexpected finding of this study is that E‐cadherin deficiency promotes and reduces the sensitivity of 2D and 3D CRCs to ferroptosis, respectively, suggesting its differential roles. It is well known that high E‐cadherin inhibits Acyl‐CoA synthetase long‐chain family 4 (ACSL4), a known promotor for ferroptosis, which enhances the ferroptosis resistance of CRCs.^[^ ^18^ ^]^ Therefore, E‐cadherin in 3D cells may trigger a multifaceted effect by simultaneously inhibiting CD61 (Figure 5H,K; Figure S11F,G, Supporting Information) and ACSL4. Since CD61 is inherently barely expressed in 3D Ctrl cells (Figure 5H), overexpression of E‐cadherin in 3D cells caused ferroptosis resistance mainly due to ACSL4 downregulation (Figure S12, Supporting Information). In addition, as a downstream target of YAP,^[^ ^18^ ^]^ ACSL4 is also subject to a dual upstream mechanism of its regulation in 3D CRCs. On the one hand, E‐cadherin inhibits ACSL4 expression through NF2‐YAP signaling,^[^ ^18^ ^]^ and on the other hand, matrix softness inhibits ACSL4 through LATS‐YAP signaling,^[^ ^29^ ^]^ so that matrix softness and low E‐cadherin expression in 3D cells play a mutual antagonistic role on ACSL4. Consistently, ACSL4 was not significantly elevated in 3D sgECAD CRCs (Figure S8A–C, Supporting Information). In this context, CD61‐mediated adhesion tension due to E‐cadherin deletion dominated the high resistance to ferroptosis in 3D sgECAD CRCs. For the mice experiments, although we standardized the tumor volume at the start of drug injection to some extent, the tumors in the 2D sgECAD vehicle group after 14 days were still much smaller than those in the 2D Ctrl vehicle group and the tumors in the 2D sgECAD group reached the size for drug injection about one week later than in the control groups (Figure S15C, Supporting Information), suggesting that E‐cadherin deficiency suppresses the growth of 2D cells in vivo. As shown in Figure S15D of the Supporting Information, E‐cadherin deletion reduced the proliferation level of 2D cells in vitro, which supported the in vivo findings. For 3D cells, E‐cadherin deletion appeared to exhibit no obvious effects on cell proliferation both in vitro (Figure S6B,C, Supporting Information) and in vivo (Figure S15C, Supporting Information). All these results suggest the differential effects of E‐cadherin deletion on cell proliferation of 2D versus 3D cells. The underlying mechanisms remain unclear, which warrants further investigation in the future.

E‐cadherin knockout in 3D CRCs induces a late hybrid EMT phenotype and enhances integrin‐mediated cell–ECM adhesion and cell membrane tension, the disruption of which diminishes the promotive effect on ferroptosis. Nevertheless, the detailed mechanisms remain unclear. In particular, the relationship between late hybrid EMT and ferroptosis defense, and the roles of cell membrane tension in late hybrid EMT warrant further investigation. Previous studies show that the absence of E‐cadherin function affects integrin expression and redistribution^[^ ^75^ ^]^ and there is crosstalk between cadherin‐dependent adhesion junctions and integrin‐based adhesion patches in response to different mechanical stimuli.^[^ ^76^ ^]^ Changes in the adhesion strength of cells may result in the tensional imbalance between these two adhesion systems, suggesting a possible link between hybrid EMT phenotype and cell membrane tension. Further, epithelial cancer cells show high membrane tension that suppresses tumor metastasis, and the induction of EMT reduces this tension and facilitates tumor cell invasion and migration.^[^ ^77^ ^]^ These findings implicate the complexity among EMT, cell mechanics, and malignancy.

In addition, the transcriptome analysis indicates the upregulation of the phospholipid‐binding protein Annexin A2 (ANXA2) in 3D E‐cadherin‐deficient cells, which has been reported to be positively associated with tumor malignancy.^[^ ^78^ ^]^ AXNA2 colocalizes with E‐cadherin, remodels cortical actin and E‐cadherin redistribution through Cofilin1, and regulates EMT through STAT3.^[^ ^79^ ^]^ Moreover, thrombin can alter the cell‐surface exposure of ANXA2,^[^ ^80^ ^]^ suggesting that 3D fibrin gels formed by fibrinogen and thrombin may have induced the aberrant expression of ANXA2 and thus affect the EMT phenotype in the absence of E‐cadherin. Therefore, the differential functions of E‐cadherin deletion in 2D and 3D CRCs may be ascribed to the combined effects of both of these biochemical and mechanical signaling.

Our findings suggest that 3D CRCs are more resistant to radiotherapy and Oxaliplatin compared to 2D cells and that knockout of E‐cadherin promotes/reduces the sensitivity of 2D/3D cells to therapy‐induced death. The inhibition of membrane tension and integrins α_v_β_3_ reduces the resistance of 3D E‐cadherin‐deficient CRCs to these therapies. These results indicate that CRCs respond to clinical treatments in a similar way to FIN‐induced ferroptosis. Since the EMT state regulates the defense ability against ferroptosis, it is thus possible that the EMT phenotype of CRC patient‐derived tumors can predict therapeutic resistance, which is supported by the findings that EMT is associated with the sensitivity of patient‐derived tumoroids to ferroptosis. Therefore, this study suggests that the EMT state of CRC tumors can serve as a promising prognostic marker to assess the therapeutic efficacy, and targeting WNT/β‐catenin signaling and integrins α_v_β_3_ may effectively overcome this resistance. A recent study shows that the morphology of pancreatic cancer organoids can anticipate the EMT status and that solid organoids are associated with partial EMT features,^[^ ^81^ ^]^ indicating the potential of tumoroids in personalized diagnosis and treatment. Together, these results suggest that the characterization of E‐cadherin/β‐catenin/CD61 expression in tumors and the drug effect of tumoroids could serve as powerful strategies for evaluating the therapeutic responses and developing potential treatment modalities for CRC precision medicine.^[^ ^82^ ^]^

## 4 Experimental Section

### Cell Culture

The human colorectal cancer cell lines HCT116 and HT29 were purchased from the National Collection of Authenticated Cell Cultures, routinely examined, and verified to be free of mycoplasma contamination. HCT116 and HT29 cells were cultured in McCoy's 5A and Roswell Park Memorial Institute (RPMI)‐1640 medium containing 10% fetal bovine serum and 1% penicillin–streptomycin in an incubator at 37 °C with 5% CO_2_.

### Mice

Half female and half male mice (4 weeks old) weighing ≈18 g were obtained from Beijing Vital River Laboratory Animal Technology Co., Ltd. The BALB/c nude mice were maintained under specific pathogen‐free conditions at the Laboratory Animal Center of the Nanjing First Hospital, Nanjing Medical University. All procedures were performed with the center's approval from the Experimental Animal Ethics Committee of Nanjing First Hospital, Nanjing Medical University (No. DWSY‐24058514).

### Biological Samples

The research protocol was reviewed and approved by the Ethics Committee of Nanjing First Hospital (No. KY20240514‐04). Clinical samples were collected from colorectal carcinoma patients who underwent surgical resection at Nanjing First Hospital, Nanjing Medical University. Following excision, a portion of the samples collected was used for organoid construction. The remaining samples were either preserved in 4% paraformaldehyde or kept at −80 °C for later research. Donors provided written, informed consent to tissue collection, analysis, and publication. Samples were numerically coded to protect donors’ rights to confidentiality and privacy. Patient information is provided in Table S1 of the Supporting Information.

### Cell Viability Assay

Cell viability was determined by CellTiter96 AQueous One Solution Cell Proliferation Assay (Promega, USA) as the instructions. 5000 cells per well were seeded in 96‐well plates one day before treatment. After being cultured with appropriate drugs, the cells were washed once with PBS. Then 20 µL CellTiter96 AQueous One Solution Reagent was pipetted into each well containing 100 µL medium and incubated for 1–4 h in an incubator at 37 °C with 5% CO_2_, the plate was read at an absorbance of 490 nm using a microplate reader (Tecan, Switzerland).

### 2D and 3D Gels Preparation

2D polyacrylamide and 3D fibrin gels were prepared following the protocol as reported previously.^[^ ^28^ ^]^ Briefly, smear a small volume of 3‐Aminopropyltrimethoxysilane (Sigma‐Aldrich, USA) evenly on the glass‐bottom dishes, covered with a film of dried NaOH. After rinsing with distilled H_2_O for 15 min twice, 0.5% glutaraldehyde was used to cover the glass. Gel stiffness was regulated by the mixture of acrylamide and bis‐acrylamide. The precursor solution was mixed with 40% acrylamide solution (Sigma‐Aldrich, USA) and 2% *N*,*N*′‐methylene bisacrylamide solution (Sigma‐Aldrich, USA) in distilled H_2_O, and gels were polymerized by adding 1/200 volume of 10% ammonium persulphate (Sigma‐Aldrich, USA) and 1/2000 volume of Tetramethylethylenediamine (Sigma‐Aldrich, USA) to precursor solution. Immediately 40 µL precursor solution was pipetted onto the preactivated glass‐bottom dishes and covered with an 18 mm coverslip. After 30 min of acrylamide polymerization, the coverslip was removed and it was washed with 100 mm HEPES. The PA gels were activated with 50 mg mL^−1^ Sulfo‐SANPAH (Sigma‐Aldrich, USA) for collagen adherence and cell seeding, and then exposed to ultraviolet radiation twice for 6 min. Following an HEPES rinse, the PA gels were incubated with 200 mg mL^−1^ of Fibrinogen (Sea Run Holdings, USA) or Collagen type I (Corning, USA) in PBS overnight at 4 °C before being washed with PBS. Cells were planted on the top of PA gels after they had been sterilized for 30 min under ultraviolet light.

To generate 3D fibrin gels, salmon fibrinogen (Sea Run Holdings, USA) was diluted with T7 buffer (50 mm Tris, 150 mm NaCl, pH 7.4). 250 µL of cells and fibrin solution were mixed with 5 µL Salmon Thrombin (Sea Run Holdings, USA) in the center of each well. The cell culture plate was placed back into the incubator after 1 mL of the medium had been supplied after 30 min. The medium on the fibrin gel was changed every 2 or 3 days.

To generate 3D collagen gels, sterile 10× PBS, 1 N NaOH, ddH_2_O, Collagen Type I (Corning, USA), and centrifuge tubes were placed on ice, and the final volume and collagen concentration were determined and calculated. 10× PBS, 1 N NaOH, ddH_2_O, and Collagen Type I were added in tubes, they were mixed thoroughly without bubbles, and placed on ice. Dilute collagen material was added to the culture surface, ensuring that the entire surface was covered. The culture plate was transferred to the cell incubator for ≈30 min.

### Immunofluorescence

Cells were seeded on the glass coverslips before, and then fixed with 4% paraformaldehyde. After permeabilization with 0.05% Triton X‐100 at room temperature for 10 min, cells were blocked by 10% donkey serum (Solarbio, China) at 4 °C for 5 h. Subsequently, the cells were incubated with primary antibodies overnight, and secondary antibodies were applied for 1 h away from light. The nucleus was identified by DAPI (BioFroxx, Germany) for 30 min. Immunofluorescence was visualized by a confocal laser scanning microscope (Carl Zeiss, Germany) or Elyra 7 with Lattice structured illumination microscopy (SIM) (Carl Zeiss, Germany).

### Electron Microscope Sample Preparation and Mitochondrial Observation

The medium was removed, the cells were gently scraped off, and they were centrifuged at 1500 rpm for 10 min, then the supernatant was discarded. 2.5% glutaraldehyde was added and fixed at 4 °C for 2 h, then fixed with 1% OsO_4_ for 30 min, and stained with 4% uranium acetate. They were then dehydrated by increasing concentrations of ethanol and propylene oxide, subsequently embedded in the resin. The 70 nm ultrathin section was mounted on a copper grid for observation and photography.

### Western Blotting

Cells were solubilized in IP or RIPA Lysis Buffer (Beyotime, China), and then the protein content was measured by the BCA Protein Assay Kit (Beyotime, China). Equal amounts of proteins were electrophoretically separated in SDS‐polyacrylamide gels and transferred to PVDF membranes (Millipore, USA). The membranes were blocked in a 5% skim milk solution for 1 h. The membranes were subsequently incubated overnight with primary antibodies at 4 °C, then with secondary antibodies for 1 h at room temperature. The antibodies used in this study are listed in Table S2 of the Supporting Information. The full set of unmodified, raw blots of western blotting data in the main figures is listed in Figure S16 of the Supporting Information.

### Real‐Time Quantitative PCR

Briefly, cells were lysed using TRIzol (Invitrogen, USA) to extract total RNA. The expression of mRNA was determined by real‐time quantitative PCR using HiScript II One Step qRT‐PCR SYBR Green Kit (Vazyme, China). The primer sequences are provided in Table S3 of the Supporting Information.

### siRNAs/Plasmids Transfection and Lentiviral Infection

siRNAs were purchased from Genepharma in China. E‐cadherin overexpression plasmids were constructed based on pCDNA3.1‐CMV‐MCS‐SV40‐Puro (Miaoling Biotechnology, China). The shECAD plasmids were generated using pLKO.1‐puro (Addgene plasmid number 8453). According to the reagent instructions, the siRNAs, shRNAs, and plasmids were transfected into cells with Lipofectamine 2000 (Invitrogen, USA).

To produce lentiviral particles, HEK293T cells were seeded at 70–80% density in a 6‐well plate, then transferred with the expression vector, packaging plasmids pMD2.Gand psPAX2 using Lipofectamine 2000. After 6 h, the medium was changed. And the virus‐containing supernatant was harvested 48 h after transfection, and clarified by filtering through 0.45 µm filters. CRCs and tumoroids were infected with the viruses in the presence of 10 µg mL^−1^ polybrene for 48 h, followed by selection with 2 µg mL^−1^ of puromycin for 3 days. At least two interfering RNAs were designed for each gene, and the one with the higher interference efficiency was selected for relevant experiments. The sequences of siRNAs and shRNAs are provided in Table S4 of the Supporting Information.

### Generation of Knockout Cells

CRISPR‐Cas9 method was used to delete E‐cadherin in HCT116 and HT‐29 cells. Single‐guide RNAs (sgRNAs) were cloned into the lentiviral vector pLentiCRISPRv2 (Miaoling Biotechnology, China) to construct plasmids. The created plasmids were subsequently transfected into HCT116 and HT29 cells. After 24 h, the cells were selected with 2 mg mL^−1^ puromycin (MedChemExpress, USA) for 2–3 days and then sorted into 96‐well plates with one cell in each well. Single clones were screened by western blotting with gene‐specific antibodies. The targeting sequences used were AAGTCACGCTGAATACAGTG, GCAATGCGTTCTCTATCCAG for E‐cadherin; TTATCGTGTGAAAGTCCGAG, CAAGGACTTCGACTTCCCGG for FAT1.

### Colony Size Assay

About 5000 colorectal tumor cells were grown in 3D fibrin gels for 6 days. Images of the colony were captured with an inverted microscope (Olympus, Japan) every 2 days. The colony volume was computed using the following formula: 4/3*π*(Area/π)^(3/2), assuming the colonies always maintain the sphere‐like shape. Surface area numbers were obtained from colony images by ImageJ software.

### Flow Cytometry

The cells were resuspended with precooled Staining Buffer (BD, USA), and the cell density was adjusted to 10^6^ cells mL^−1^. 100 µL cell suspension was transferred into a round‐bottomed polypropylene tube, and then an appropriate amount of antibody was added to each tube and incubated at room temperature for 30 min in the dark. The cells were washed twice with Staining Buffer. Fluorescence was assessed by Beckman flow cytometry, then analyzed and plotted using the FlowJo software.

### Mitotracker, MitoPY1, Bodipy, and Hoechst Staining

Mitotracker (ThermoFisher SCIENTIFIC, USA), MitoPY1 (TOCRIS, UK), BODIPY 581/591 C11 (ThermoFisher SCIENTIFIC, USA), and Hoechst 33342 (Apexbio, USA) were added directly to the cell culture medium already in the culture yielding a final concentration of 200 nm for 1 h, 5 and 20 µm for 30 min before live cell imaging. Three to five images per condition were analyzed using ImageJ software.

### Metabolomics Sample Preparation and Liquid Chromatography‐Mass Spectrometry (LC‐MS) Analysis

2D and 3D CRCs were collected and mixed with 1 mL sample solvent (methanol:acetonitrile:water, 2:2:1). After subjected to 3 freeze–thaw cycles, samples were incubated at −20 °C for 1 h, following centrifugation at 12000 rpm for 15 min at 4 °C. Supernatants were transferred to clean tubes and dried in a vacuum evaporator. The dry metabolite extracts were reconstituted into 100 µL solvent (acetonitrile:water, 2:1), vortexed for 30 s, sonicated for 5 min at 4 °C, centrifuged at 12000 rpm for 15 min at 4 °C, and then supernatants were collected into a fresh sampler vial. Prepared samples were analyzed by LC‐MS. Metabolites were identified by presence of signal at both MRM transitions and by comparing to the retention times of pure standards and the levels of gamma‐glutamylcysteine were quantified from raw peak areas normalized to total biomass. Finally, the peak areas were used for comparative quantification.

### Quantification of Intracellular GSH

No less than 5 million cells were collected, PBS resuspension cells were added, and 600 × *g* was subsequently centrifuged for 10 min. 1 mL Reagent One was added to suspend the cells, and then ultrasound (200 W, ultrasound 3 s, stopping for 10 s, repeated 30 times). The supernatant was obtained by centrifugation at 8000 × *g* for 10 min. The absorbance of 412 nm was determined and calculated according to the Reduced Glutathione (GSH) Content Assay Kit's instructions (Solarbio, China).

### Measurement of Intracellular Ferrous Ions and ROS

Cells were stained with FerroOrange (Dojindo, Japan, final concentration of 5 µm) or H2DCFDA (Dojindo, Japan, final concentration of 1 µm) for 30 min following treatments. The cells were then collected and washed once with PBS, followed by flow cytometry or fluorescence microscope analysis in cold PBS. Fluorescence in live cells was captured by a fluorescence microscope or plotted using the FlowJo software.

### ChIP Assay

The ChIP assay was performed following the SimpleChIP Enzymatic Chromatin IP Kit's guidelines (Cell Signaling Technology, USA). Briefly, cells under different treatments were fixed with formaldehyde, and the chromatin was digested with micrococcal nuclease to form 1–5 chromatin fragments. Then the lysate was immunoprecipitated with β‐catenin or TCF4 antibody. Immunoprecipitated DNAs were analyzed by RT‐qPCR. The ChIP primers are listed in Table S3 of the Supporting Information.

### Dual‐Luciferase Reporter Gene Assay

The full‐length coding sequences of *β‐catenin* and *TCF4* were inserted into the pCDNA3.1(+) vector (Keygen, China) as effectors, while the promoter sequences of *GPX1*, *GPX4*, *FTH1*, and *FTL* were fused upstream of the firefly luciferase reporter gene of pGL3‐basic vector (Keygen, China) as reporters. HCT116 cells were seeded in a 6‐well plate and transfected with 1 µg of each of the luciferase reporters + 100 ng Renilla‐luciferase plasmid pRL‐TK (Keygen, China) for normalization. The promoterless luciferase vector was used to assess the background. After 24 h, cells were digested and reseeded on 2D or in 3D 24‐well plates. Cells were then lysed and Firefly and Renilla Luciferase activities were measured using the Dual Luciferase Reporter Gene Assay Kit (YEASEN, China). The relative luciferase activities are the mean of at least three independent experiments carried out in duplicate or triplicate.

### Measurement of Cell Membrane Tension

Cell tension was measured by FLIM using Fluorescence Lifetime Probe Flipper‐TR (Cytoskeleton, USA). The probe specifically targets the cytoplasmic membrane and reflects changes in membrane tension by reporting changes in fluorescence lifetime. Cells were inoculated in Confocal Petri dishes and stained using Flipper‐TR for 20 min. Fluorescence lifetime values of cell membrane tension were acquired by Leica Sp8 FLIM microscope and calculated by LAS X FLIM/FCS with n‐Exponential Reconvolution Fitting. Phasor FLIM analysis further provided a 2D graphical view of lifetime distributions.

### Xenograft Experiments

The xenograft experiments were performed by a protocol approved by the Experimental Animal Ethics Committee of Nanjing First Hospital, Nanjing Medical University. The study is compliant with all relevant ethical regulations regarding animal research. Cancer cells under different treatments were resuspended in PBS and injected into nude mice subcutaneously. Tumor volumes and nude mice weight were measured every 2 days. Once tumors reached a diameter of ≈5 mm, mice were treated with vehicle (diluted solution: 5% DMSO + 40% PEG300 + 5% Tween80 + 50% ddH_2_O or ddH_2_O), IKE (50 mg kg^−1^), LF3 (50 mg kg^−1^), Cilengitide (100 µg per 100 µL ddH_2_O) or combination regimens by intraperitoneal injection every two days for 14 days. The tumor volume was quantified using the formula of length times the square of width times 0.52. The length was also considered as the diameter here. The maximal tumor burden permitted by the ethics committee is a length of 1.5 cm, and the maximal tumor burden did not exceed the limit. Tumor tissues were collected at 4 to 5 weeks after injection.

### Immunohistochemistry and Multiplex IHC

Animal tumor tissues or biological samples from humans were fixed with 4% paraformaldehyde, then paraffin‐embedded and sectioned. The slides were deparaffinization, rehydration, and antigen retrieval before being blocked with 5% BSA (Sigma‐Aldrich, USA) for 1 h. The primary antibodies were then incubated with the slides overnight at 4 °C. The slides were treated with 3,3′‐Diaminobenzidine (Sigma‐Aldrich, USA) and hematoxylin (Sigma‐Aldrich, USA) after the secondary antibodies were incubated for 30 min at room temperature. Images of immunohistochemistry were obtained at random under a microscope, and the outcomes were examined using ImageJ software.

For multiplex IHC, the slides were deparaffinization, rehydration, and antigen retrieval before being blocked with 10% donkey serum for 30 min. The primary antibodies were then incubated with the slides overnight at 4 °C. The slides were treated with Tyramine salt‐CY3/CY5/488/594/450 sequentially after the secondary antibodies (HRP enzyme labeled) were incubated for 50 min at room temperature. Images of mIHC were obtained at random under a fluorescence microscope. Nuclei stained with DAPI were blue under UV excitation and positive signals were labeled with the corresponding fluorescein. Positive signal intensity in the tumor tissue area was quantified separately for each section using the Indica Labs – Area Quantification FL v2.1.2 module in Halo v3.0.311.314 analysis software.

### Tumoroid Culture

The tissue was rinsed three times with 1× D‐PBS, which contains 2% antibiotics. Using surgical scissors or scalpels, the tissue was minced into little pieces measuring 1–3 mm^3^. The tissue fragments were digested with Gentle Cell Dissociation Reagent (Stemcell, USA) at 37 °C for 30 min. DMEM/F12 medium (KeyGEN Biotechnology, China) was added to stop digestion, and was filtered utilizing a 70 µm cell strainer. The filtered cells were collected and centrifuged at 300 × *g* for 5 min at 4 °C. The supernatant was aspirated and the pellet was resuspended in Matrigel basement membrane matrix (Corning, USA). The amount of Matrigel used depends on the size of the pellet. Approximately 10 000 cells should be plated in 25 µL of Matrigel. Droplets of ≈30 µL each were placed around the center of the well, and then the culture plate was placed into an incubator at 37 °C and 5% CO_2_ for 15–20 min to let the Matrigel solidify. Once the Matrigel droplets were solidified, 500 µL of organoid complete medium was added to each well.

### Tumoroid Viability Assay

Tumoroids were harvested and seeded at 100–200 crypts per well in a 2:3 mixture of Matrigel and Colorectal Cancer Organoid Kit (bioGenous, China) in 96‐well plates. On day 3, medium was removed and fresh medium containing varying concentrations of drugs (IKE, LF3, and Cilengitide) was added at three replicates per drug dose. The CellTiter96 AQueous One Solution Cell Proliferation Assay (Promega, USA) was used to assess the viability of the tumoroids as the instructions. Tumoroid‐free Matrigel (10 µL) served as a control. Optical density was measured at an absorbance of 490 nm using a microplate reader (Tecan, Switzerland).

### Measurement of Tumoroid Viability, Bodipy, PI, and ROS

Tumoroid viability was measured using the CellTiter96 AQueous One Solution Cell Proliferation Assay (Promega, USA) according to the manufacturer's instructions. Bodipy, PI, and ROS levels were measured using BODIPY581/591 C11 (ThermoFisher SCIENTIFIC, USA), Calcein‐AM/PI (Solarbio, China), and Reactive Oxygen Species Assay Kit (Solarbio, China). Briefly, tumoroids were treated as indicated with different concentrations of IKE and stained, then analyzed by fluorescence microscopy and ImageJ.

### Transcriptome Sequencing and Bioinformatics Analysis

The sequencing of transcriptome and bioinformatics analysis was performed using Illumina Novaseq 6000 by Lianchuan Biotechnology in China. The twofold change in gene transcription expression and a *P*‐value of less than 0.05 are the screening requirements for differential genes. The obtained differential genes were subjected to KEGG enrichment analysis using the tools^[^ ^83^ ^]^ at https://www.omicstudio.cn/tool; https://www.kegg.jp/kegg/rest/keggapi.html. The correlation analysis between EMT markers and ferroptosis regulaters was analyzed using R. All the ferroptosis regulators were extracted from the FerrDb database.^[^ ^84^ ^]^

### Statistical Analysis

Statistical analyses of bar graphs and scatter plots in this paper were performed using GraphPad Prism or Microsoft Excel. Statistical significance between two groups was determined by two‐tailed Student's *t*‐test, and that for more than two groups was determined by the ordinary one‐way ANOVA or two‐way ANOVA with Tukey's multiple comparison test. All of the statistical details of experiments can be found in the figures and figure legends. Data from at least three independent experiments (*n* = 3) were presented as Means ± SEMs. *P* values of less than 0.05 were considered significant.

## Conflict of Interest

The authors declare no competing interests

## Author Contributions

X.W., Y.G., Y.Z., and S.Z. contributed equally to this work. Q.J. conceived the project. L.H., Y.T., and Q.J. designed the experiments. X.W., Y.G., Y.Z., S.Z., Y.Z., Y.C., N.W., X.W., J.Z., X.Z., L.H., Y.T., and Q.J. carried out experiments and performed analyses. X.W., Y.G., Y.Z., S.Z., L.H., Y.T., and Q.J. all participated in writing the manuscript.

## Supporting information



Supporting Information

Supplemental Table 1

## Data Availability

RNA‐seq datasets generated during this study are available at NCBI Gene Expression Omnibus (GEO) under accession code GSE186662, GSE186731, and GSE240744.
