# Supplementary material for: Hybrid EMT Phenotype and Cell Membrane Tension Promote Colorectal Cancer Resistance to Ferroptosis
Source: Adv Sci (Weinh). 2025 Feb 22;12(15):2413882. doi: 10.1002/advs.202413882 (PMC12005738; doi:10.1002/advs.202413882)
Supplement: Supplementary file 1 — Supporting Information [file ADVS-12-2413882-s001.pdf]

ADVANCED SCIENCE
Open Access

Supporting Information

for *Adv. Sci.*, DOI 10.1002/advs.202413882

Hybrid EMT Phenotype and Cell Membrane Tension Promote Colorectal Cancer Resistance to Ferroptosis

Xiaowei Wei, Yutong Ge, Yaolin Zheng, Sunyan Zhao, Yuhan Zhou, Yuhan Chang, Nuofan Wang, Xiumei Wang, Juan Zhang, Xuanchang Zhang, Liqiao Hu, Youhua Tan* and Qiong Jia**

Supporting information

Hybrid EMT phenotype and cell membrane tension promote colorectal cancer resistance to ferroptosis

Xiaowei Wei[1$], Yutong Ge[1,2$], Yaolin Zheng[3$], Sunyan Zhao[1$], Yuhan Zhou[1], Yuhan Chang[4], Nuofan Wang[5], Xiumei Wang[1], Juan Zhang[1], Xuanchang Zhang[1], Liqiao Hu[6*], Youhua Tan[7*] and Qiong Jia[1*]

*Correspondence:
hu_liqiao@gzlab.ac.cn (L.H.)
youhua.tan@polyu.edu.hk (Y.T.)
jiaqiong2020@njmu.edu.cn (Q.J.)

This file includes:

Supplementary Figure. S1 to S16

Legends of Table S1-S5

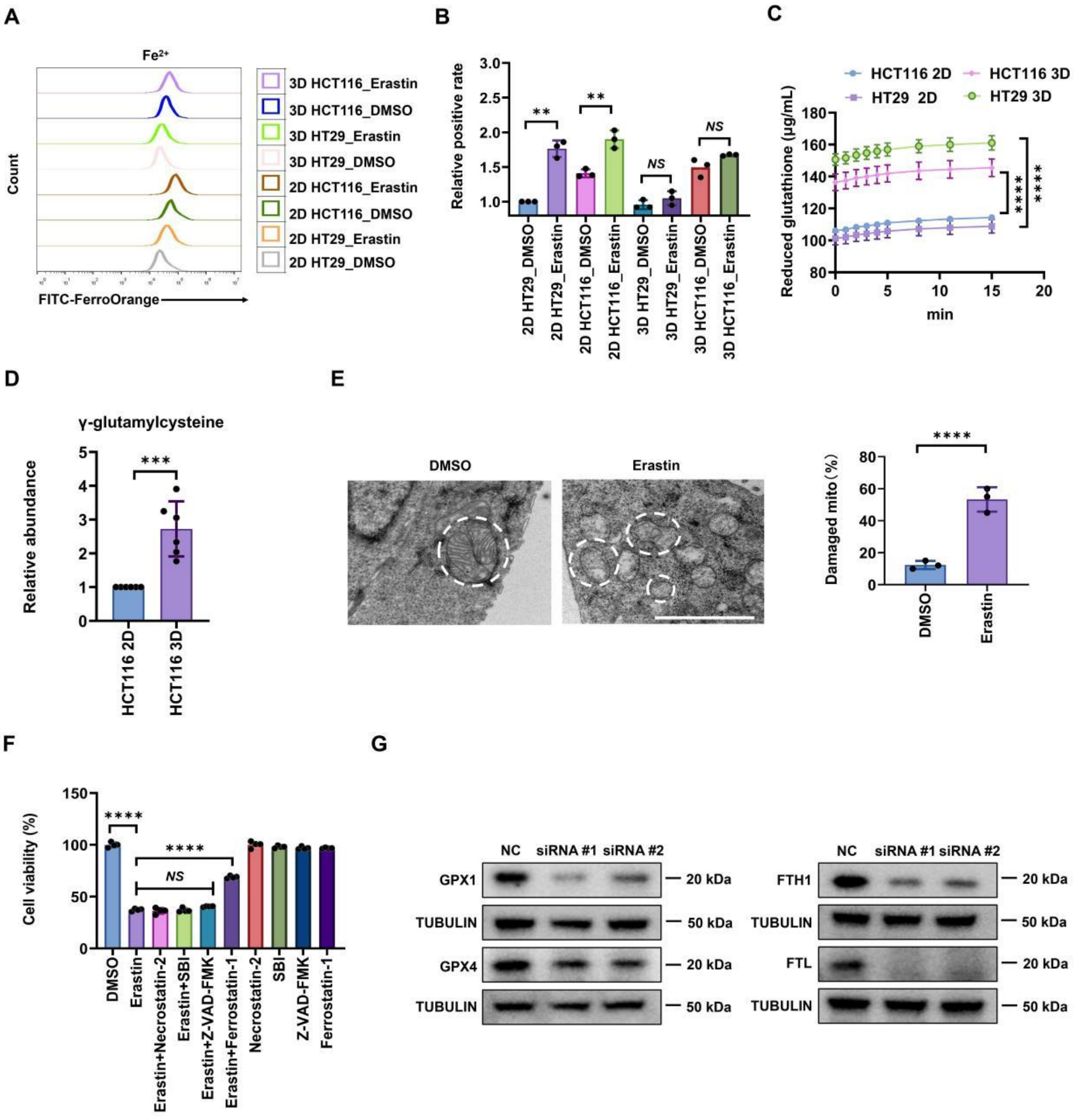

Figure S1 Glutathione peroxidase and ferritin are involved in ferroptosis resistance of 3D CRCs

A and B. 2D and 3D CRCs treated with DMSO or 15 μM Erastin for 24 hours. The level of ferrous irons was determined by flow cytometry using a FerroOrange probe. Means ± SEMs; n = 3. $^{**}P < 0.01$. ***NS***, non-significant difference analyzed by two-tailed Student's *t*-test.

C. Reduced Glutathione (GSH) concentration in 2D and 3D CRCs cultured for 3 days was detected. Means ± SEMs; n = 3. $^{****}P < 0.0001$ analyzed by two-tailed Student's *t*-test.

D. HCT116 cells were cultured on 2D or in 3D for 3 days. The levels of

gamma-glutamylcysteine were detected by LC-MS. Means ± SEMs; n = 6. ***P < 0.001 analyzed by two-tailed Student's *t*-test.

E. Transmission electron microscopy of HCT116 cells treated with DMSO or 15 μM Erastin for 24 hours. The white dashed circles indicate the location of mitochondria. Scale bar, 2 μm. A minimum of 3 cells per treatment condition were examined for mitochondria morphology. Means ± SEMs; n = 3. ****P < 0.0001 analyzed by the ordinary one-way ANOVA with Tukey's multiple comparison test.

F. Relative cell viability of 2D HCT116 cells treated with Erastin 10 μM for 24 hours after pretreatment by different known small molecule cell death inhibitors for 6 hours (5 μM Ferrostatin-1, 2 μM Necrostatin-2, 1 μM SBI, 5 μM Z-VAD-FMK). Means ± SEMs; n = 4. ***NS***, non-significant difference among all the indicated groups, ****P < 0.0001 analyzed by the ordinary one-way ANOVA with Tukey's multiple comparison test.

G. 2D HCT116 cells were transfected with negative control (**NC**) or siRNAs targeting *GPX1*, *GPX4*, *FTH1 or FTL*. After 24 hours, total proteins were extracted to detect the knockdown efficiency of siRNAs. n = 3.

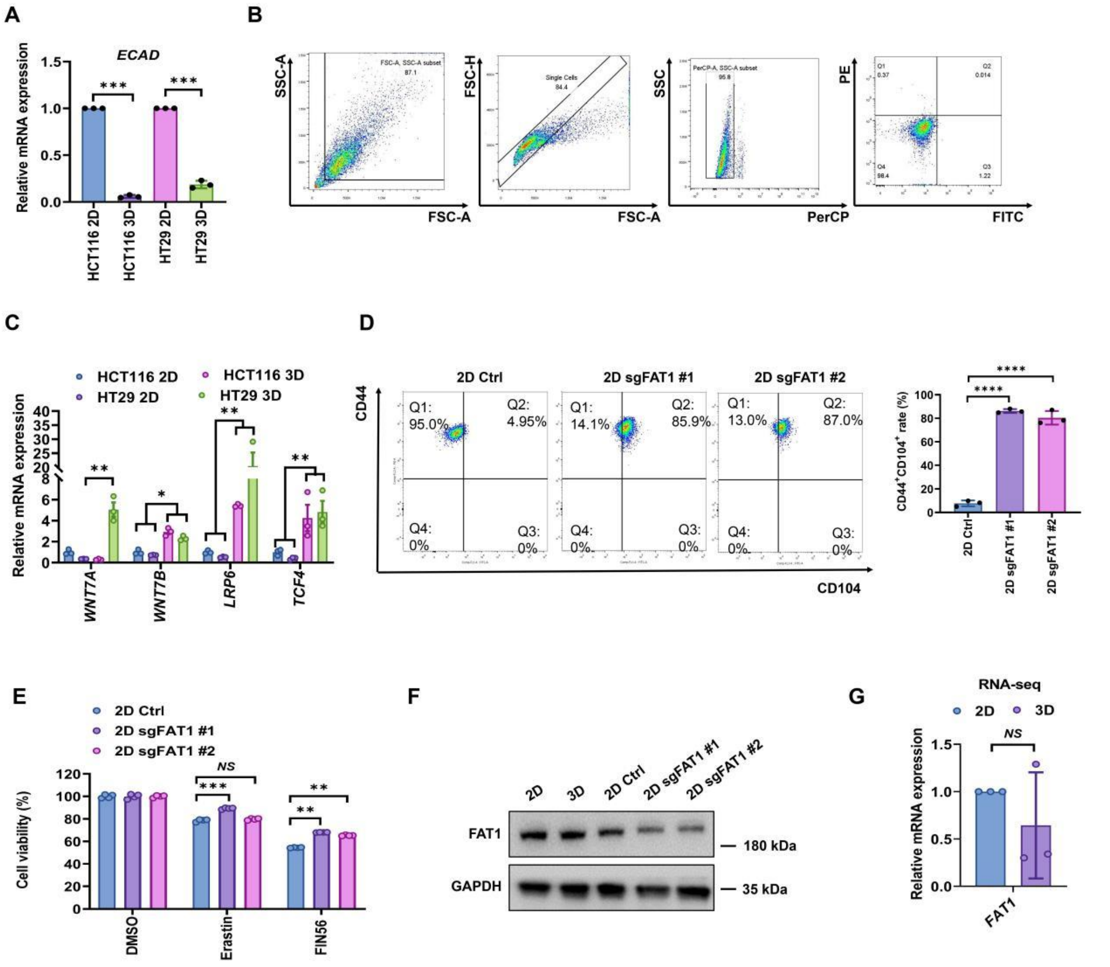

Figure S2 3D culture and substrate softness contribute to the hybrid EMT phenotype of 3D CRCs

A. The mRNA expression of *E-cadherin* (*ECAD)* in 3D cells relative to 2D cells. Means ± SEMs; n = 3. ***$P < 0.001$ analyzed by two-tailed Student's *t*-test.

B. Gating strategy for FACS analysis. The FACS image first displays the gating strategy used to isolate live single cells for analyzing surface protein immunostaining, followed by a cross gate to distinguish between CD104/CD44 negative or positive cell populations.

C. Total mRNA was extracted from 2D and 3D cells, and qPCR was performed to detect the expression of *WNT7A*, *WNT7B*, *LRP6* and *TCF4*. Means ± SEMs; n = 3. *$P < 0.05$, **$P < 0.01$ analyzed by two-tailed Student's *t*-test.

D. Flow cytometry profiles for CD104 and CD44 of 2D cells transfected with

non-targeting control (**Ctrl**), or sgRNAs targeting FAT1 (sgFAT1). Means ± SEMs; n = 3. ****P < 0.0001 analyzed by the ordinary one-way ANOVA with Tukey's multiple comparison test.

E. 2D Ctrl and 2D sgECAD cells were transfected with Ctrl or sgRNA targeting FAT1, and then treated with DMSO, 10 µM Erastin or 4 µM FIN56 for 24 hours. Relative cell viability of cells was detected. Means ± SEMs; n = 4. ***NS***, non-significant difference, **P < 0.01, ***P < 0.001 analyzed by the ordinary one-way ANOVA with Tukey's multiple comparison test.

F. FAT1 and GAPDH protein levels in 2D, 3D, and 2D sgFAT1 cells were detected by western blotting. n = 3. Relative mRNA expression of FAT1 extracted from RNA-seq data. Means ± SEMs; n = 3. ***NS***, non-significant difference.

G. FAT1 mRNA expressions of 2D and 3D CRC were extracted from RNA-seq data. Means ± SEMs; n = 3. ***NS***, non-significant difference analyzed by two-tailed Student's *t*-test.

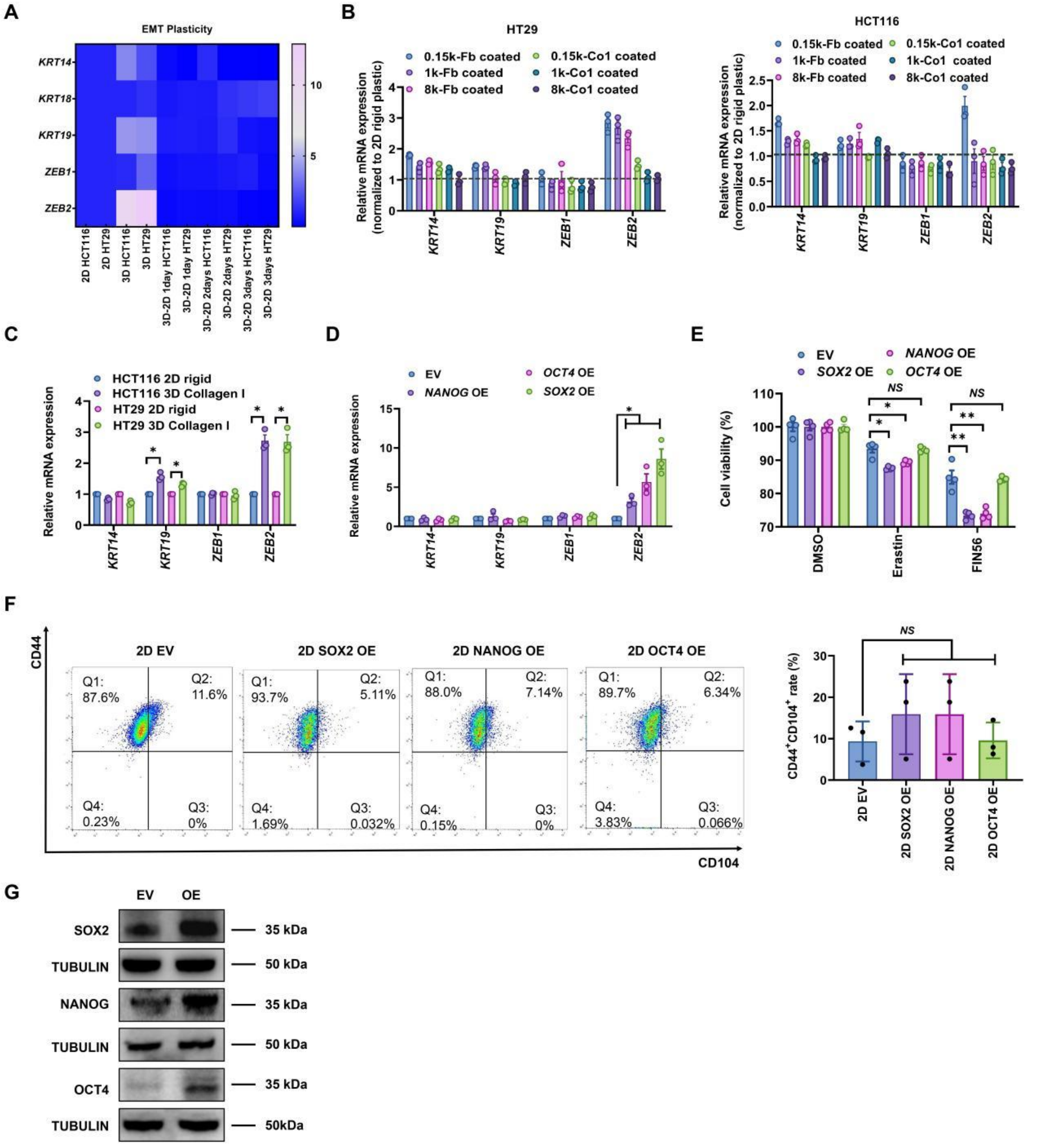

Figure S3 Matrix softness determined stemness sustained the mesenchymal phenotype of CRCs

A. 2D or 3D CRCs were collected and replated on rigid plastic for 1, 2 and 3 days, respectively. Total mRNA was extracted, and qPCR was performed to detect the expression of EMT markers such as *KRT14*, *KRT18*, *KRT19*, *ZEB1* and *ZEB2*. Means ± SEMs; n = 3.

B. Cells were cultured on 2D PA gels coated with Fibrinogen (**Fb)** or Collagen I (**Co1**), with varying rigidity (0.15, 1, 8 kPa) respectively. After 24 hours, Total mRNA

was extracted, and qPCR was performed to detect the expression of *KRT14*, *KRT19*, *ZEB1* and *ZEB2*. mRNA expressions of cells cultured on 2D rigid plastic (6-well plate) were set at 1, indicated by the black dashed line. Means ± SEMs; n = 3.

C. CRCs were cultured on 2D rigid plastic or in 3D collagen I gels for 3 days. Total mRNA was extracted, and qPCR was performed to detect the expression of *KRT14*, *KRT19*, *ZEB1* and *ZEB2*. Means ± SEMs; n = 3. **P* < 0.05 analyzed by two-tailed Student's *t*-test.

D. 2D HT29 cells were transfected with EV, *NANOG*, *SOX2* or *OCT4* OE plasmid for 24 hours. Total mRNA was extracted to detect the expression of *KRT14*, *KRT19*, *ZEB1* and *ZEB2*. Means ± SEMs; n = 3. **P* < 0.05 analyzed by the ordinary one-way ANOVA with Tukey's multiple comparison test.

E. 2D HT29 cells were transfected with EV, *NANOG*, *SOX2* or *OCT4* OE plasmid, then treated with DMSO, 10 μM Erastin or 4 μM FIN56 for 24 hours. The relative cell viability of 2D cells was detected. Means ± SEMs; n = 4. ***NS***, non-significant difference, **P* < 0.05, ***P* < 0.01 analyzed by the ordinary one-way ANOVA with Tukey's multiple comparison test.

F. Flow cytometry profiles for CD104 and CD44 of 2D cells transfected with EV, *NANOG*, *SOX2* or *OCT4* OE plasmid. Means ± SEMs; n = 3. ***NS***, non-significant difference analyzed by the ordinary one-way ANOVA with Tukey's multiple comparison test.

G. 2D HT29 cells were transfected with EV, *SOX2*, *NANOG* or *OCT4* OE plasmid. After 24 hours, total protein was extracted to detect the overexpression efficiency. n = 3.

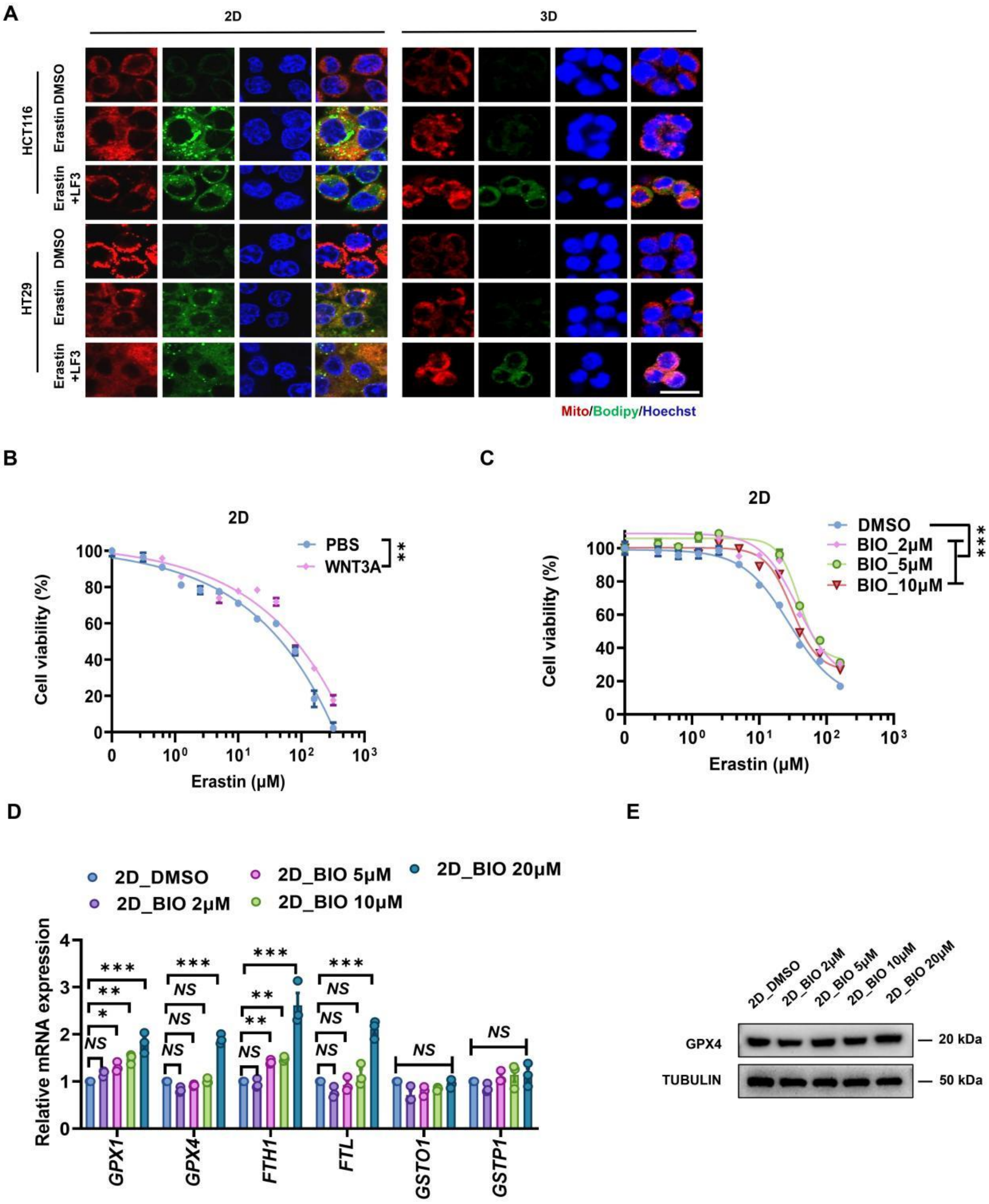

Figure S4 WNT-GPXs/ferritin axis mediates ferroptosis defense of 3D CRCs

A. 2D and 3D CRCs were pretreated with DMSO or 5 µM LF3 for 12 hours and then treated with 15 µM Erastin for 24 hours. The cells were stained with a BODIPY-C11 probe (**green**), Mito-tracker (**red**), and Hoechst (**blue**) respectively. Scale bar, 20 µm. At least 3 views were randomly selected for each condition.

B. Relative cell viability of 2D HCT116 cells treated with different concentrations of

Erastin for 24 hours after pretreatment by WNT3A protein (100 μg/ml) for 12 hours. Means ± SEMs; n = 4. **P < 0.01 analyzed by two-tailed Student's *t*-test.

C. Relative cell viability of 2D HCT116 cells treated with different concentrations of Erastin for 48 hours after pretreatment by GSK-3 inhibitor BIO (2, 5, 10 μM) for 12 hours. Means ± SEMs; n = 4. **P < 0.01 analyzed by the ordinary one-way ANOVA with Tukey's multiple comparison test.

D and E. 2D HCT116 cells were treated with different concentrations of GSK-3 inhibitor BIO (2, 5, 10, 20 μM) for 24 hours. Total mRNA and protein were extracted to detect the expression of GPX1, GPX4, GSTP1, GSTO1, FTH1 and FTL. Means ± SEMs; n = 3. ***NS***, non-significant difference, *P < 0.05, **P < 0.01, ***P < 0.001 analyzed by the ordinary one-way ANOVA with Tukey's multiple comparison test.

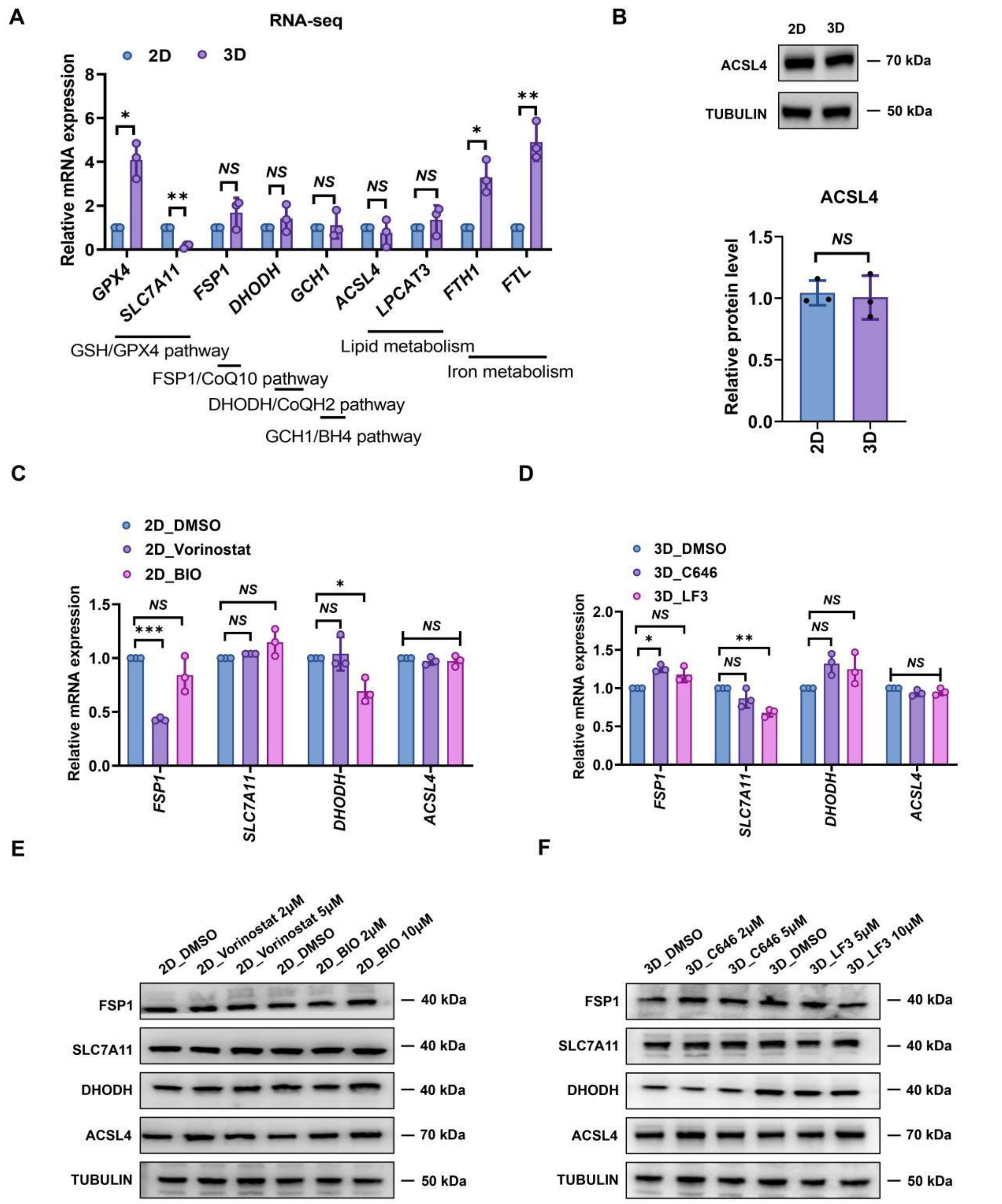

Figure S5 Global analysis of differential ferroptosis regulatory pathways in 2D and 3D CRCs

A. Relative mRNA expressions of the indicated ferroptosis regulators extracted from RNA-seq data. Means ± SEMs; n = 3. ***NS***, non-significant difference, **P* < 0.05, ***P* < 0.01 analyzed by two-tailed Student's *t*-test.

B. Western blotting analysis of 2D and 3D cultured HCT116 cells for ACSL4 and

TUBULIN. Relative protein levels of each protein were normalized to TUBULIN. Means ± SEMs; n = 3. ***NS***, non-significant difference analyzed by two-tailed Student's *t*-test.

C. 2D HCT116 cells were treated with DMSO, 5 μM Vorinostat or 5 μM BIO for 24 hours. Total mRNA was extracted to detect the expression of *FSP1*, *SLC7A11*, *DHODH*, and *ACSL4*. Means ± SEMs; n = 3. ***NS***, non-significant difference, **P* < 0.05, ***P* < 0.01, ****P* < 0.001 analyzed by the ordinary one-way ANOVA with Tukey's multiple comparison test.

D. 3D HCT116 cells were treated with DMSO, 5 μM C646 or 5 μM LF3 for 24 hours. Total mRNA was extracted to detect the expression of *FSP1*, *SLC7A11*, *DHODH*, and *ACSL4*. Means ± SEMs; n = 3. ***NS***, non-significant difference, **P* < 0.05, ***P* < 0.01, ****P* < 0.001 analyzed by the ordinary one-way ANOVA with Tukey's multiple comparison test.

E. 2D HCT116 cells were treated with DMSO, Vorinostat or BIO for 24 hours. Total protein was extracted to detect the expression of FSP1, SLC7A11, DHODH, and ACSL4. n = 3.

F. 3D HCT116 cells were treated with DMSO, C646 or LF3 for 24 hours. Total protein was extracted to detect the expression of FSP1, SLC7A11, DHODH, and ACSL4. n = 3.

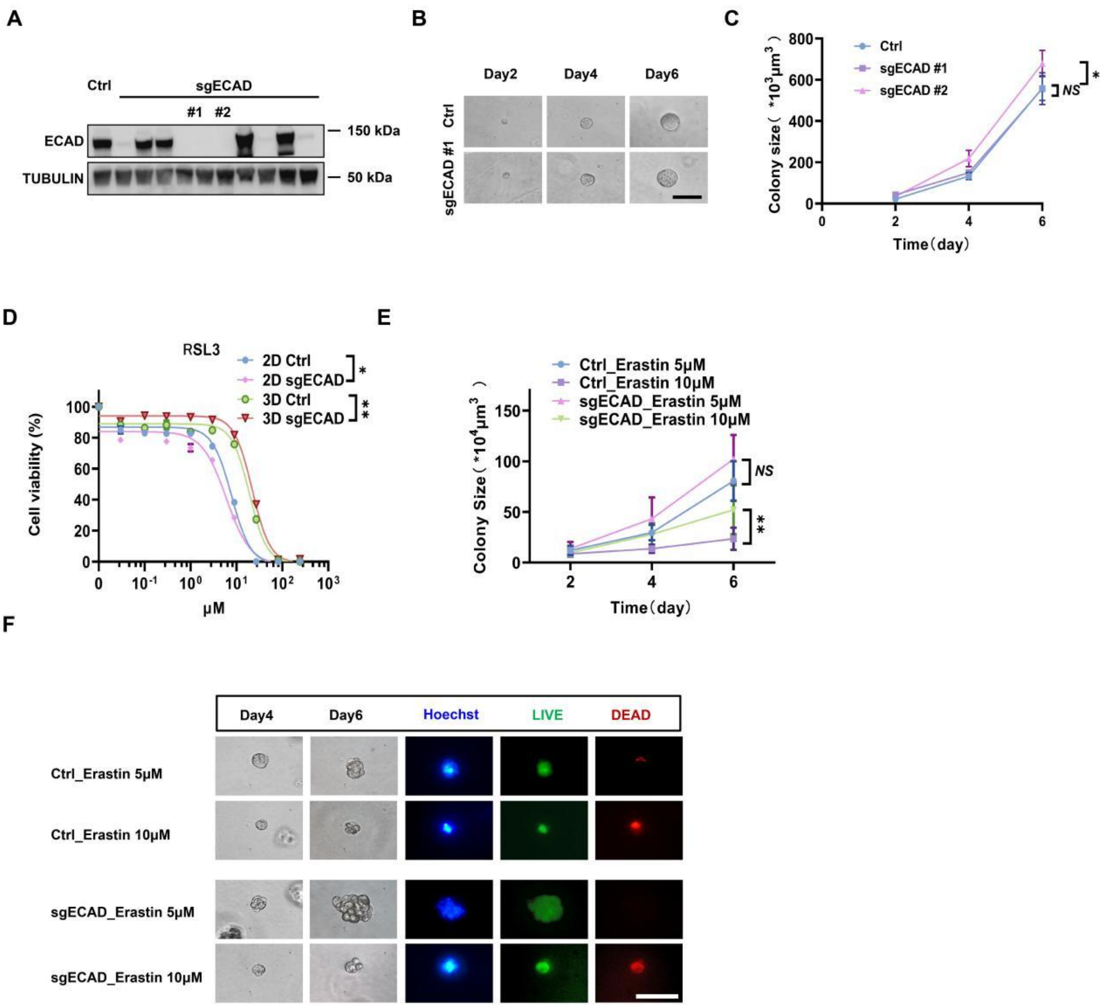

Figure S6 E-cadherin-deficient 3D CRCs exhibit super resistance to ferroptosis

A. HT29 cells were transfected with non-targeting control (**Ctrl**), or sgRNAs targeting E-cadherin (**sgECAD**) using CRISPR-cas9 technology. GAPDH and ECAD protein expression levels of Ctrl and sgECAD cells were determined by western blotting. n = 3.

B and C. Ctrl, sgECAD #1 and sgECAD #2 cells were cultured in 3D for 6 days. Colony morphology was captured every 2 days, scale bar, 200 μm; colony size (volume) was recorded every 2 days. Means ± SEMs; n = 15 randomly selected colonies for each condition. ***NS***, non-significant difference, **P* < 0.05 analyzed by the ordinary one-way ANOVA with Tukey's multiple comparison test.

D. Relative cell viability of 2D Ctrl, 2D sgECAD, 3D Ctrl, and 3D sgECAD cells treated with different concentrations of RSL3 for 24 hours. Means ± SEMs; n = 4. ***NS***,

non-significant difference, *P < 0.05, **P < 0.01 analyzed by the ordinary one-way ANOVA with Tukey's multiple comparison test.

E and F. 3D Ctrl and 3D sgECAD cells were treated with 5 μM or 10 μM Erastin for 6 days. Colony morphology was captured every 2 days. ***NS***, non-significant difference, **P < 0.01 analyzed by the ordinary two-way ANOVA with Tukey's multiple comparison test. Cells were stained with propidium iodide (PI) for dead cells (**red**), Calcein-AM for living cells (**green**) and Hoechst for nuclei (**blue**) on day 6. Scale bar, 200 μm. At least 3 colonies were randomly selected for each condition.

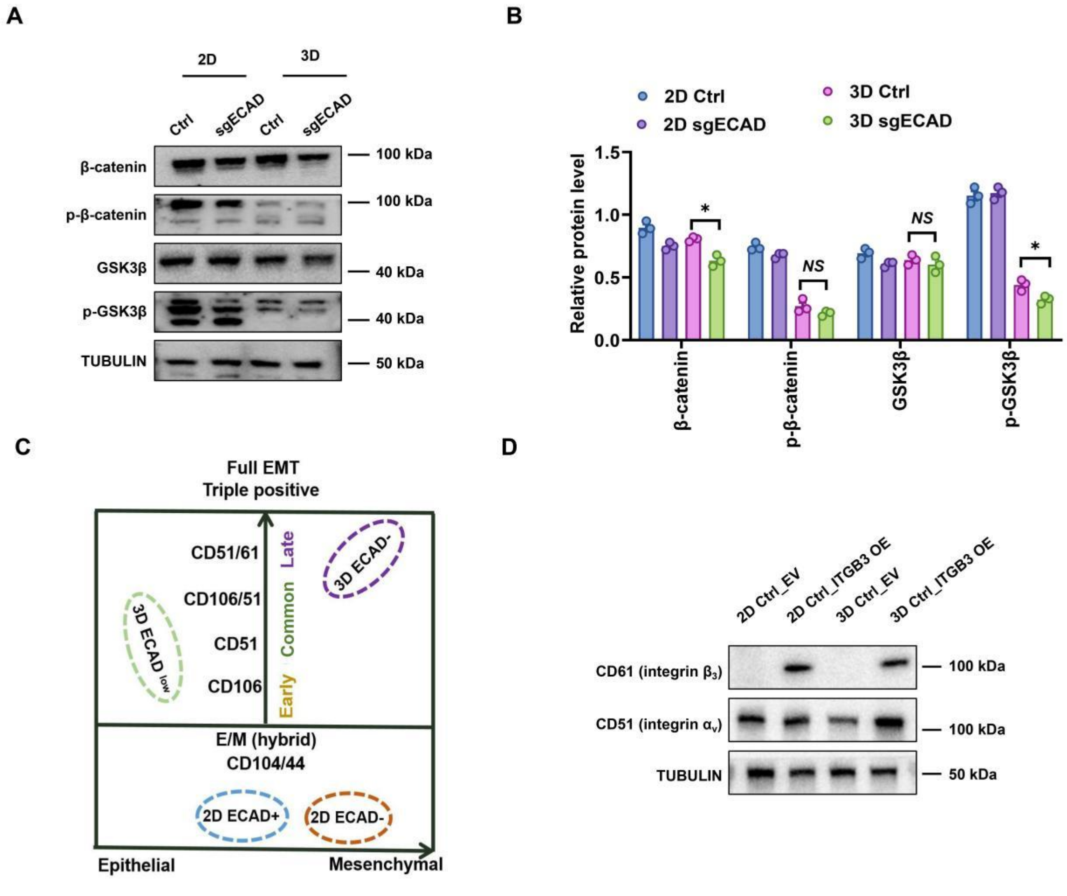

Figure S7 E-cadherin deletion in 3D CRC induces a CD61-labeled late hybrid EMT state

A and B. Phospho-β-catenin, total β-catenin, phospho-GSK3β, total GSK3β and TUBULIN protein levels in 2D Ctrl, 2D sgECAD, 3D Ctrl, and 3D sgECAD cells

cultured for 3 days were detected by western blotting. Relative protein levels of each protein were normalized to TUBULIN. Means ± SEMs; n = 3. ***NS***, non-significant difference, *$P < 0.05$ analyzed by the ordinary one-way ANOVA with Tukey's multiple comparison test.

C. A schematic diagram illustrated the distinct EMT states defined by CD44, CD104, CD106, CD51, and CD61 for 2D and 3D cells with different levels of ECAD (ref. 7).

D. 2D or 3D Ctrl cells were transfected with EV or ITGB3 OE plasmid. CD61, CD51 and TUBULIN protein levels were determined by western blotting. n = 3.

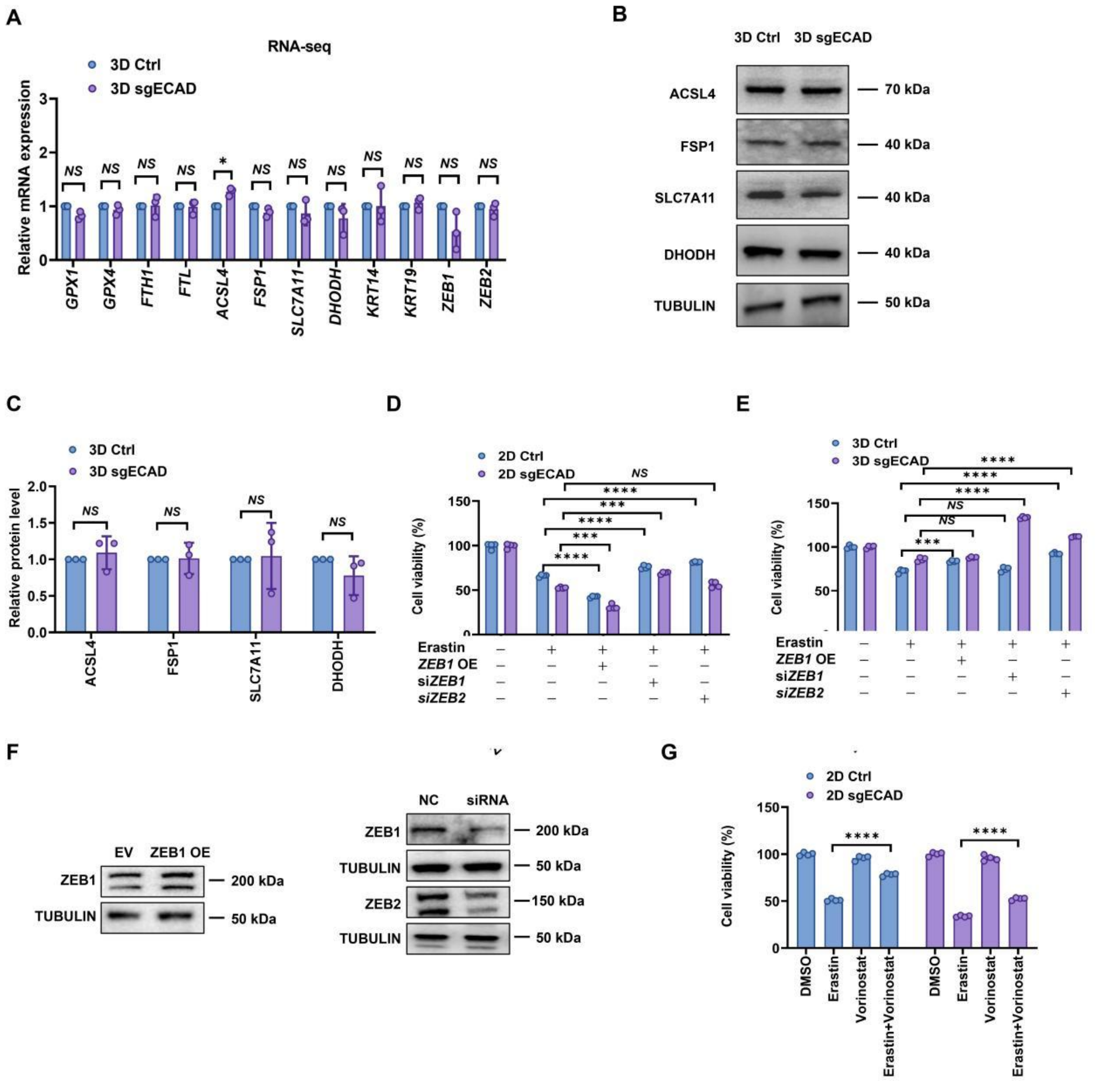

Figure S8 Known ferroptosis regulators or EMT-associated molecules may not explain the enhanced ferroptosis defense of 3D E-cadherin-deficient cells

A. Relative mRNA expressions of the indicated ferroptosis and EMT-associated genes extracted from RNA-seq data. Means ± SEMs; n = 3. ***NS***, non-significant difference, *$P < 0.05$ analyzed by two-tailed Student's *t*-test.

B and C. FSP1, SLC7A11, DHODH, ACSL4, and TUBULIN protein levels in 3D Ctrl, and 3D sgECAD cells cultured for 3 days were detected by western blotting. Relative protein levels of each protein were normalized to TUBULIN. Means ± SEMs; n = 3. ***NS***, non-significant difference analyzed by two-tailed Student's *t*-test.

D. 2D Ctrl and 2D sgECAD cells were transfected with NC, *ZEB1/ZEB2* siRNAs or *ZEB1* OE plasmid, then treated with DMSO or 15 μM Erastin for 24 hours. The relative cell viability of 2D Ctrl and 2D sgECAD cells was detected. Means ± SEMs; n = 4. ***NS***, non-significant difference, ***$P < 0.001$, ****$P < 0.0001$ analyzed by the ordinary one-way ANOVA with Tukey's multiple comparison test.

E. 3D Ctrl or 3D sgECAD cells were transfected with NC, *ZEB1/ZEB2* siRNAs or *ZEB1* OE plasmid, then treated with DMSO or 15 μM Erastin for 24 hours. The relative cell viability of 3D Ctrl and 3D sgECAD cells was detected. Means ± SEMs; n = 4. ***NS***, non-significant difference, ***$P < 0.001$, ****$P < 0.0001$ analyzed by the ordinary one-way ANOVA with Tukey's multiple comparison test.

F. 2D Ctrl cells were transfected with NC, *ZEB1/ZEB2* siRNAs or *ZEB1* OE plasmid. After 24 hours, total protein was extracted to detect the knockdown efficiency. n = 3.

G. 2D Ctrl and 2D sgECAD cells were pretreated with 1 μM Vorinostat for 6 hours and then treated with DMSO or 10 μM Erastin for 24 hours. Relative cell viability of cells was detected. Means ± SEMs; n = 4. ****$P < 0.0001$ analyzed by the ordinary one-way ANOVA with Tukey's multiple comparison test.

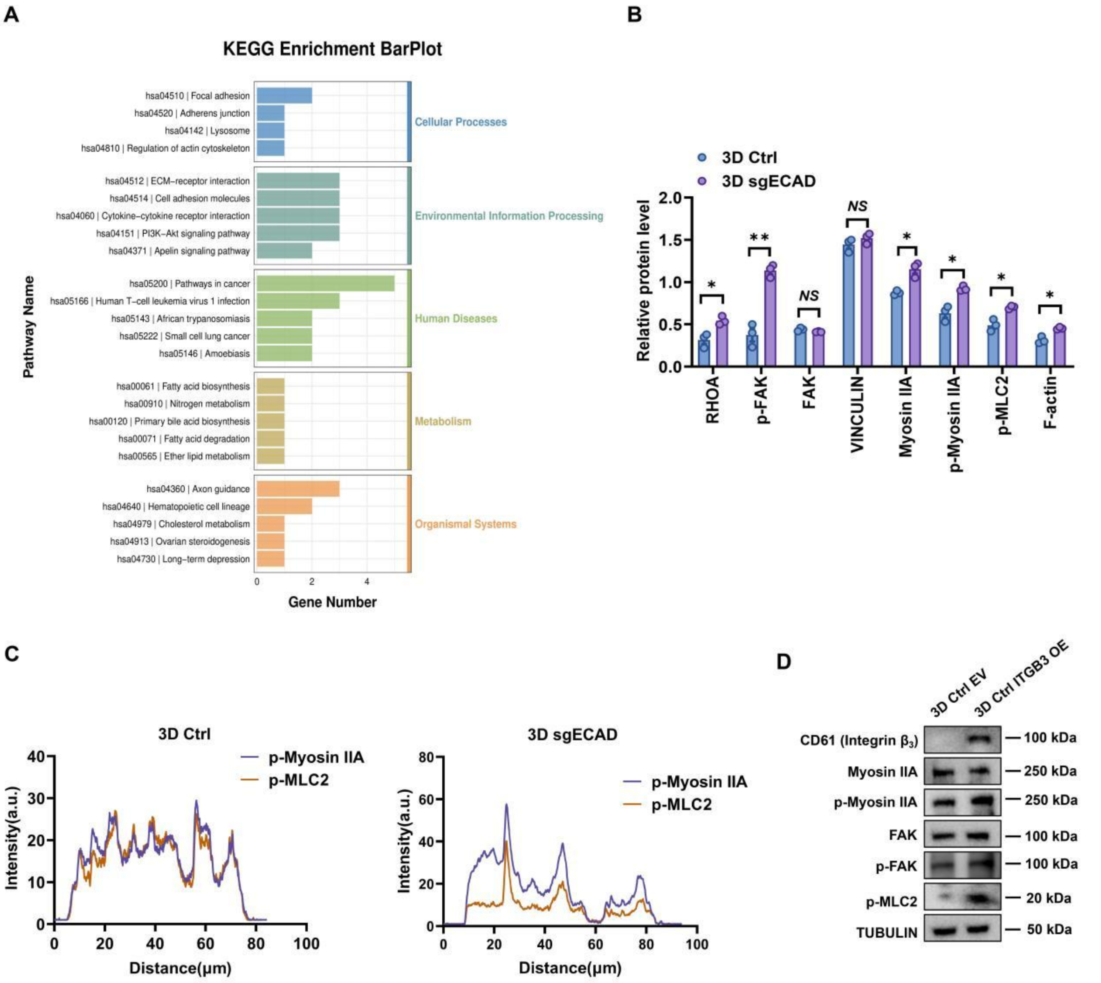

Figure S9 E-cadherin deficiency in 3D CRCs activates the proteins associated with actomyosin-mediated cell tension and focal adhesion

A. KEGG enrichment of 3D Ctrl and 3D sgECAD transcripts. n = 3.

B. Quantitative analysis of protein levels for RHOA, phospho-FAK, FAK, VINCULIN, Myosin IIA, phospho-Myosin IIA, phospho-MLC2, F-actin in 3D Ctrl and 3D sgECAD CRCs by western blotting. Relative protein levels of each protein were normalized to TUBULIN. Means ± SEMs; n = 3. ***NS***, non-significant difference analyzed by two-tailed Student's *t*-test.

C. The fluorescence intensity and co-localization of p-MLC2 and p-Myosin IIA in 3D Ctrl and 3D sgECAD cells were quantified by ImageJ software Plot Profile plugin.

D. 3D Ctrl cells were transfected with EV or ITGB3 OE plasmid. CD61, Myosin IIA, phospho-Myosin IIA, FAK, phospho-FAK, phospho-MLC2 and TUBULIN protein

levels were determined by western blotting. n = 3.

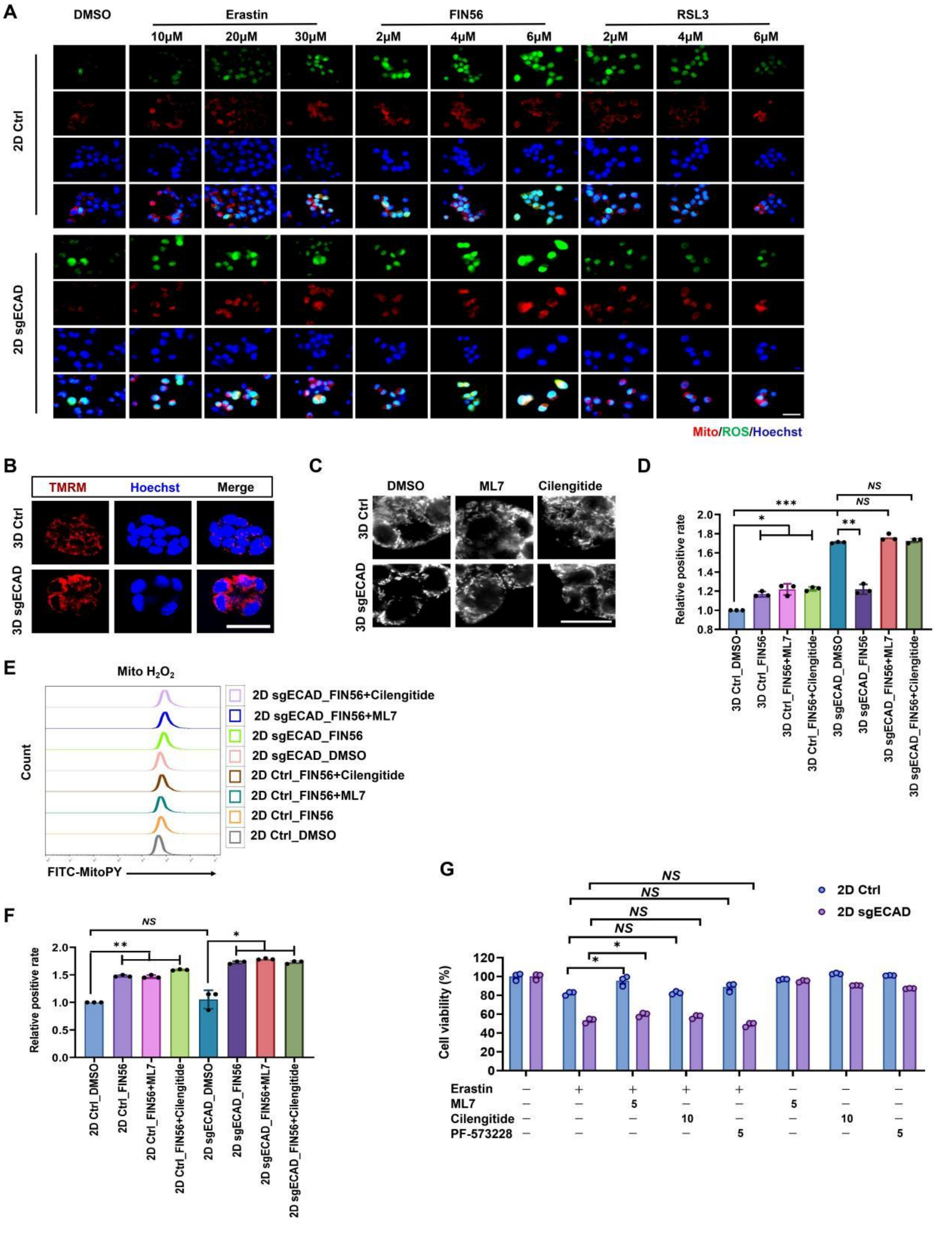

Figure S10 Cell-ECM adhesion-forced mitochondrial reprogramming counteracts ferroptosis by altering OxSR

A. 2D Ctrl and 2D sgECAD cells were treated with DMSO, Erastin (10, 20, 30 μM),

FIN56 (2, 4, 6 μM), or RSL3 (2, 4, 6 μM) for 24 hours. Cells were stained with an H2DCFDA probe (**green**), Mito-tracker (**red**) and Hoechst (**blue**) respectively by imaging using a fluorescence microscope. Scale bar, 20 μm. At least 3 views were randomly selected for each condition.

B. The mitochondrial membrane potential of 3D Ctrl and 3D sgECAD cells was imaged with confocal microscopy after being stained with 20 nM Tetramethylrhodamine (TMRM) for 30 min. Scale bar, 20 μm. At least 3 views were randomly selected for each condition.

C. 3D Ctrl and 3D sgECAD cells were treated with DMSO, smooth muscle myosin light chain kinase (MLCK) inhibitor ML7 (10 μM), and integrin αvβ3 selective inhibitor Cilengitide (10 μM) for 24 hours. Confocal Microscopy depicting mitochondrial network structure in 3D Ctrl and 3D sgECAD cells stained with 200nM Mitotracker. Scale bar, 20 μm. At least 3 views were randomly selected for each condition.

D. 3D Ctrl and 3D sgECAD cells were pretreated with DMSO, 10 μM ML7 and 10 μM Cilengitide for 6 hours and then treated with DMSO or 4 μM FIN56 for 24 hours. Mitochondrial hydrogen peroxide level of 3D Ctrl and 3D sgECAD cells was measured with flow cytometry after being stained with 5 μM mitoPY-1 for 1 hour. Means ± SEMs; n = 3. ***NS***, non-significant difference. *$P < 0.05$, **$P < 0.01$, ***$P < 0.001$ analyzed by the ordinary one-way ANOVA with Tukey's multiple comparison test.

E and F. 2D Ctrl and 2D sgECAD cells were pretreated with DMSO, 10 μM ML7 and 10 μM Cilengitide for 6 hours, and then treated with DMSO or 4 μM FIN56 for 24 hours. Mitochondrial hydrogen peroxide (Mito H_2O_2) level of 2D Ctrl and 2D sgECAD cells was measured with flow cytometry after being stained with 5 μM mitoPY-1 for 1 hour. Means ± SEMs; n = 3. ***NS***, non-significant difference. *$P < 0.05$, **$P < 0.01$ analyzed by the ordinary one-way ANOVA with Tukey's multiple comparison test.

G. 2D Ctrl and 2D sgECAD cells were pretreated with DMSO, 10 μM Cilengitide for 6 hours, 5 μM ML7, or 5 μM PF-573228 for 24 hours and then treated with DMSO or

15 μM Erastin for 24 hours. Relative cell viability of cells was detected. Means ± SEMs; n = 4. ***$P < 0.001$ analyzed by the ordinary one-way ANOVA with Tukey's multiple comparison test.

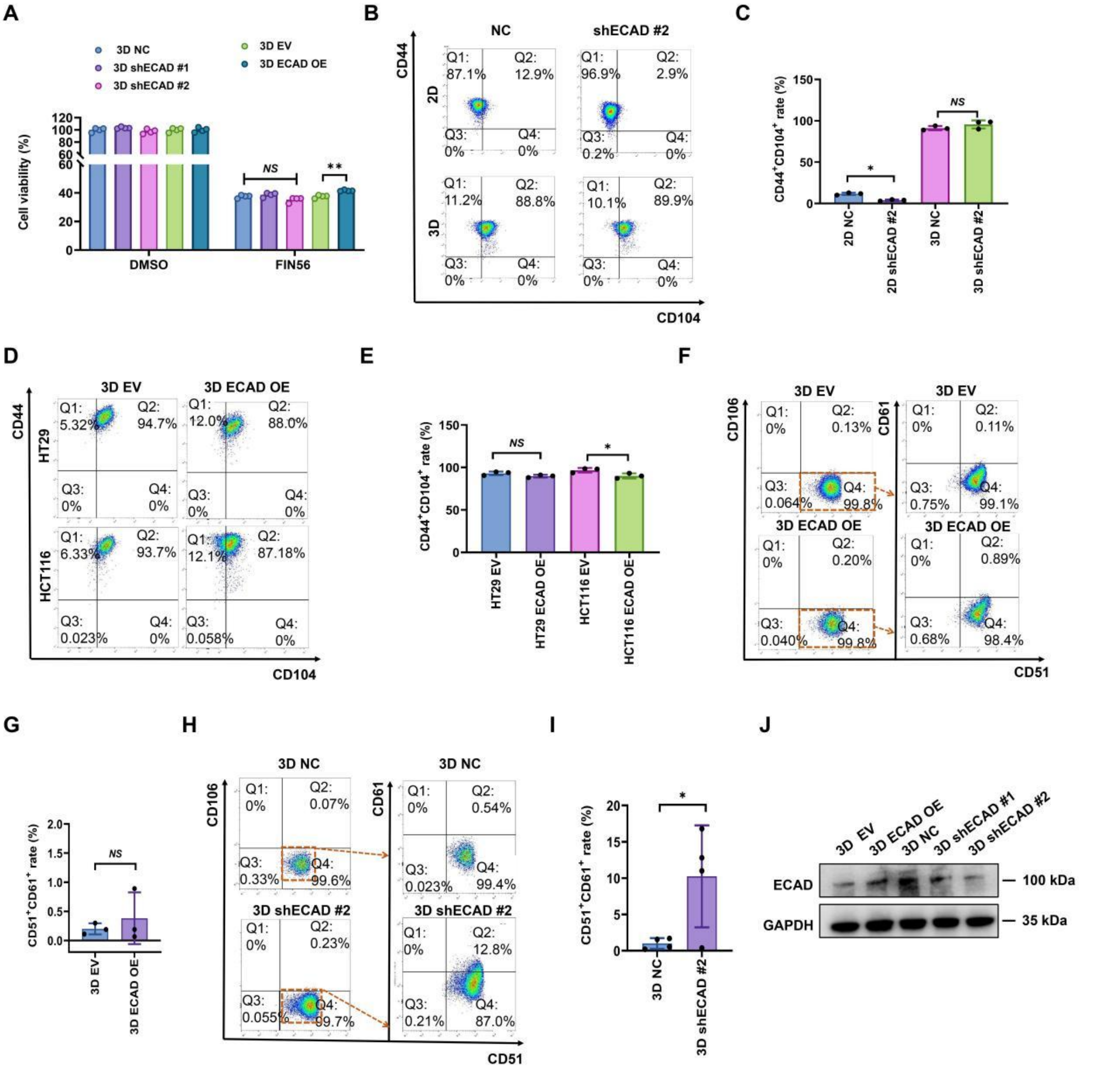

Figure S11 Knowdown or overexpression of E-cadherin has little effect on the ferroptosis sensitivity of 3D CRCs

A. 3D CRCs were infected with the viruses of scrambled (**NC**) or *ECAD* shRNAs, EV or *ECAD* OE plasmid, then treated with DMSO or 4 μM FIN56 for 24 hours. The relative cell viability of 3D cells was detected. Means ± SEMs; n = 4. ***NS***, non-significant difference, **$P < 0.01$ analyzed by the ordinary one-way ANOVA with Tukey's multiple comparison test.

B and C. Flow cytometry profiles for CD104 and CD44 of 3D CRCs infected with the viruses of scrambled or *ECAD* shRNA #2. Means ± SEMs; n = 3. ***NS***, non-significant difference, *$P < 0.05$ analyzed by the ordinary one-way ANOVA with Tukey's multiple comparison test.

D and E. Flow cytometry profiles for CD104 and CD44 of 3D CRCs infected with the viruses of EV or *ECAD* OE plasmid. Means ± SEMs; n = 3. ***NS***, non-significant difference, *$P < 0.05$ analyzed by two-tailed Student's *t*-test.

F and G. Flow cytometry profiles for CD106, CD51 and CD61 of 3D CRCs infected with the viruses of EV or *ECAD* OE plasmid. Means ± SEMs; n = 3. ***NS***, non-significant difference, *$P < 0.05$ analyzed by two-tailed Student's *t*-test.

H and I. Flow cytometry profiles for CD106, CD51 and CD61 of 3D CRCs infected with the viruses of scrambled or *ECAD* shRNA #2. Means ± SEMs; n = 3. *$P < 0.05$ analyzed by two-tailed Student's *t*-test.

J. 3D CRCs were infected with the viruses of scrambled (**NC**) or *ECAD* shRNAs, EV or *ECAD* OE plasmid. After 48 hours, total protein was extracted to detect the interference efficiency. n = 3.

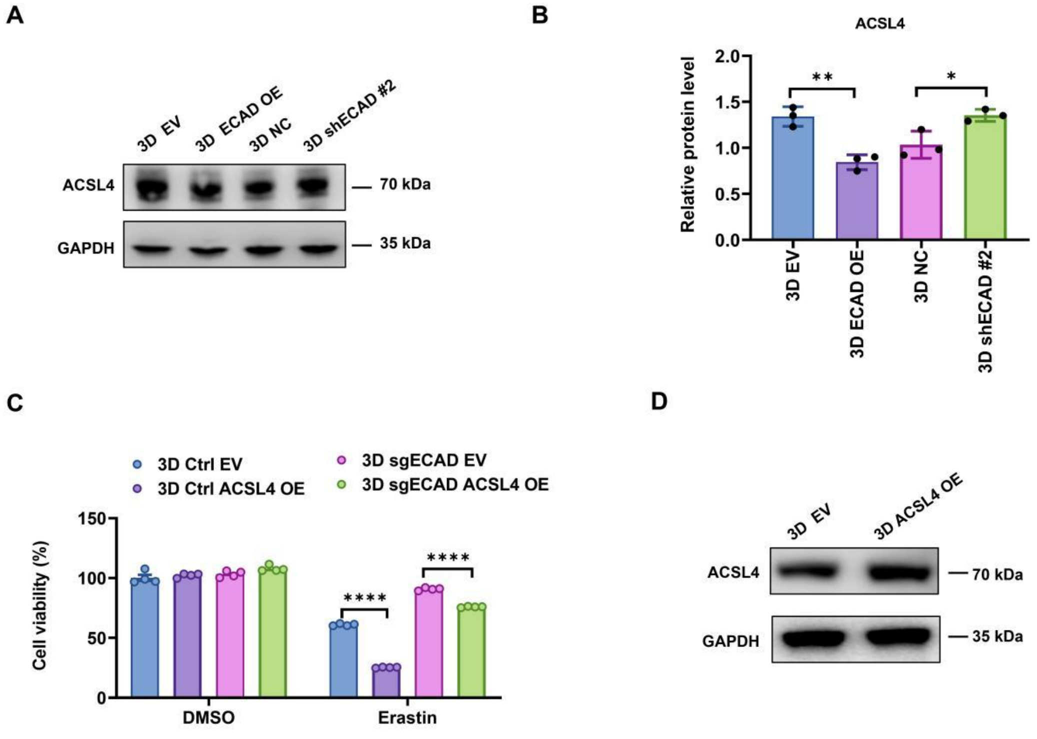

Figure S12 E-cadherin expression influences ferroptosis sensitivity of 3D CRCs

through negative regulation of ACSL4

A and B. 3D CRCs were infected with the viruses of EV or *ECAD* OE, scrambled (**NC**) or *ECAD* shRNA#2. After 48 hours, total protein was extracted to detect ACSL4 expression. Relative protein levels of ACSL4 were normalized to GAPDH. Means ± SEMs; n = 3. $*P < 0.05$, $**P < 0.01$ analyzed by two-tailed Student's *t*-test.

C. 3D Ctrl or 3D sgECAD CRCs were infected with the viruses of EV or *ACSL4* OE plasmid, then treated with DMSO or 4 μM FIN56 for 24 hours. The relative cell viability of 3D cells was detected. Means ± SEMs; n = 4. ***NS***, non-significant difference, $****P < 0.0001$ analyzed by the ordinary one-way ANOVA with Tukey's multiple comparison test.

D. 3D CRCs were infected with the viruses of EV or *ACSL4* OE plasmid. After 48 hours, total protein was extracted to detect the overexpression efficiency. n = 3.

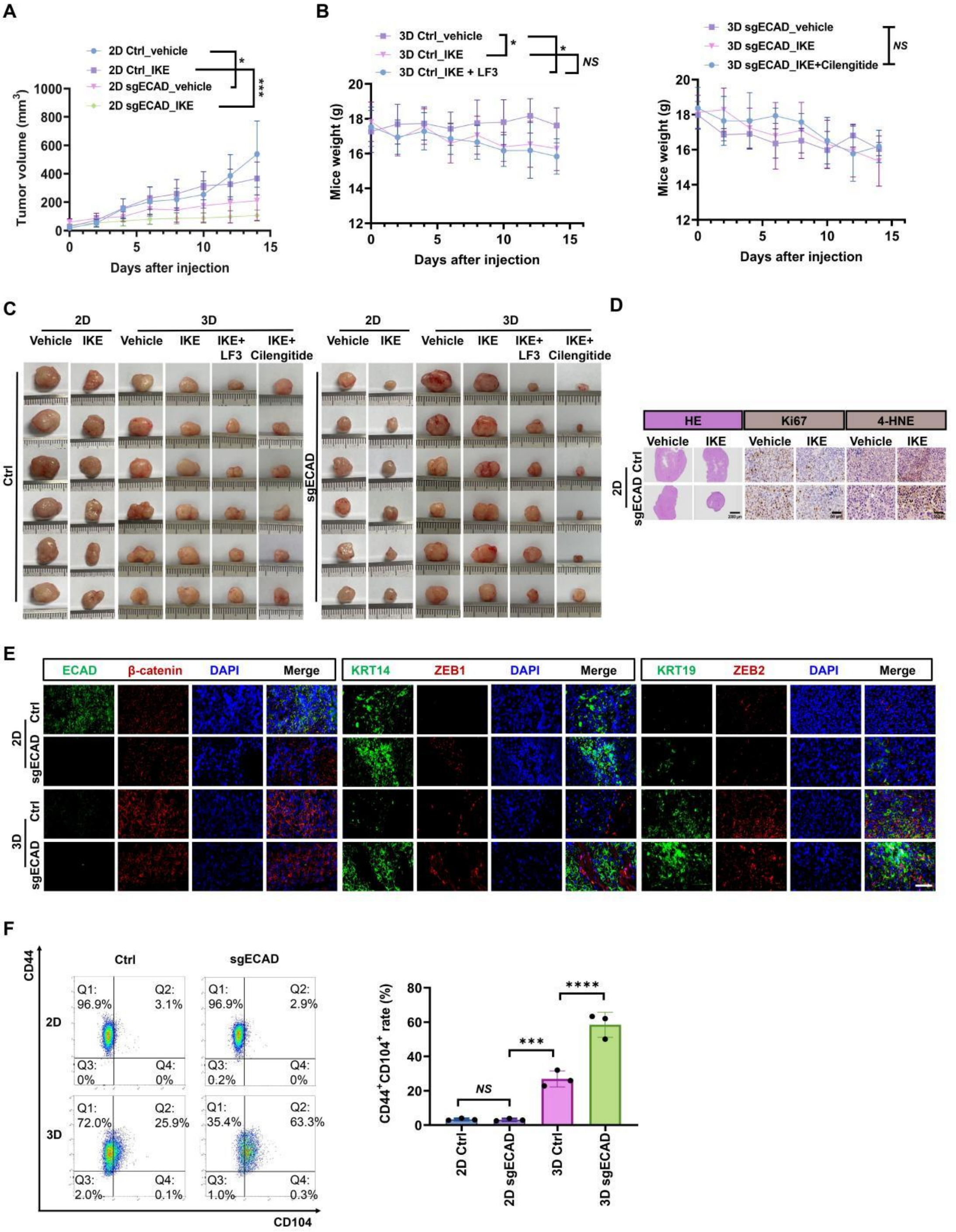

Figure S13 Mice experiments verify the distinct contributions of EMT states to CRC ferroptosis

A. Statistical analysis of the 2D Ctrl or sgECAD tumor volumes treated with vehicle or IKE (50 mg/kg) by intraperitoneal injection every two days for 14 days. At least 6 mice for each group. Means ± SEMs; **P* < 0.05, ****P* < 0.001 analyzed by the ordinary two-way ANOVA with Tukey's multiple comparison test.

B. Body weight analysis of the mice with 3D Ctrl or sgECAD tumors treated with vehicle, IKE (50 mg/kg), IKE+LF3 (50 mg/kg), or IKE+Cilengitide (100 μg) by intraperitoneal injection every two days for 14 days. At least 6 mice for each group. Means ± SEMs; ***NS***, non-significant difference, *$P < 0.05$ analyzed by the ordinary two-way ANOVA with Tukey's multiple comparison test.

C. Images of tumor tissue from different mice. Cells were subcutaneously injected into BALB/c nude mice after adjusting the cell concentration to 10^6. Once the tumors reached a diameter of around 5 mm, mice were treated with vehicle, IKE (50 mg/kg), IKE+LF3 (50 mg/kg), or IKE+Cilengitide (100 μg) by intraperitoneal injection every two days for 14 days. Six tumors were shown for each group.

D. Haematoxylin and eosin (HE) (scale bar, 2000 μm), Ki-67 and 4HNE staining (scale bar, 50 μm) of 2D Ctrl and 2D sgECAD tumor tissues of mice treated with vehicle, IKE (50 mg/kg) or IKE+LF3 (50 mg/kg). At least 3 views were randomly selected for each condition.

E. Tumor tissue sections from different mice were fixed and stained with ECAD (**green**) and β-catenin (**red**), KRT14 (**green**) and ZEB1(**red**), KRT19 (**green**) and ZEB2 (**red**) antibodies. Nuclei were visualized by DAPI (**blue**). Scale bar, 100 μm. At least 3 views were randomly selected for each group.

F. Flow cytometry profiles for CD104 and CD44 of tumor tissue digestion mixture from different mice. n = 3. Means ± SEMs; ***NS***, non-significant difference, ***$P <$ 0.001, ****$P < 0.0001$ analyzed by the ordinary two-way ANOVA with Tukey's multiple comparison test.

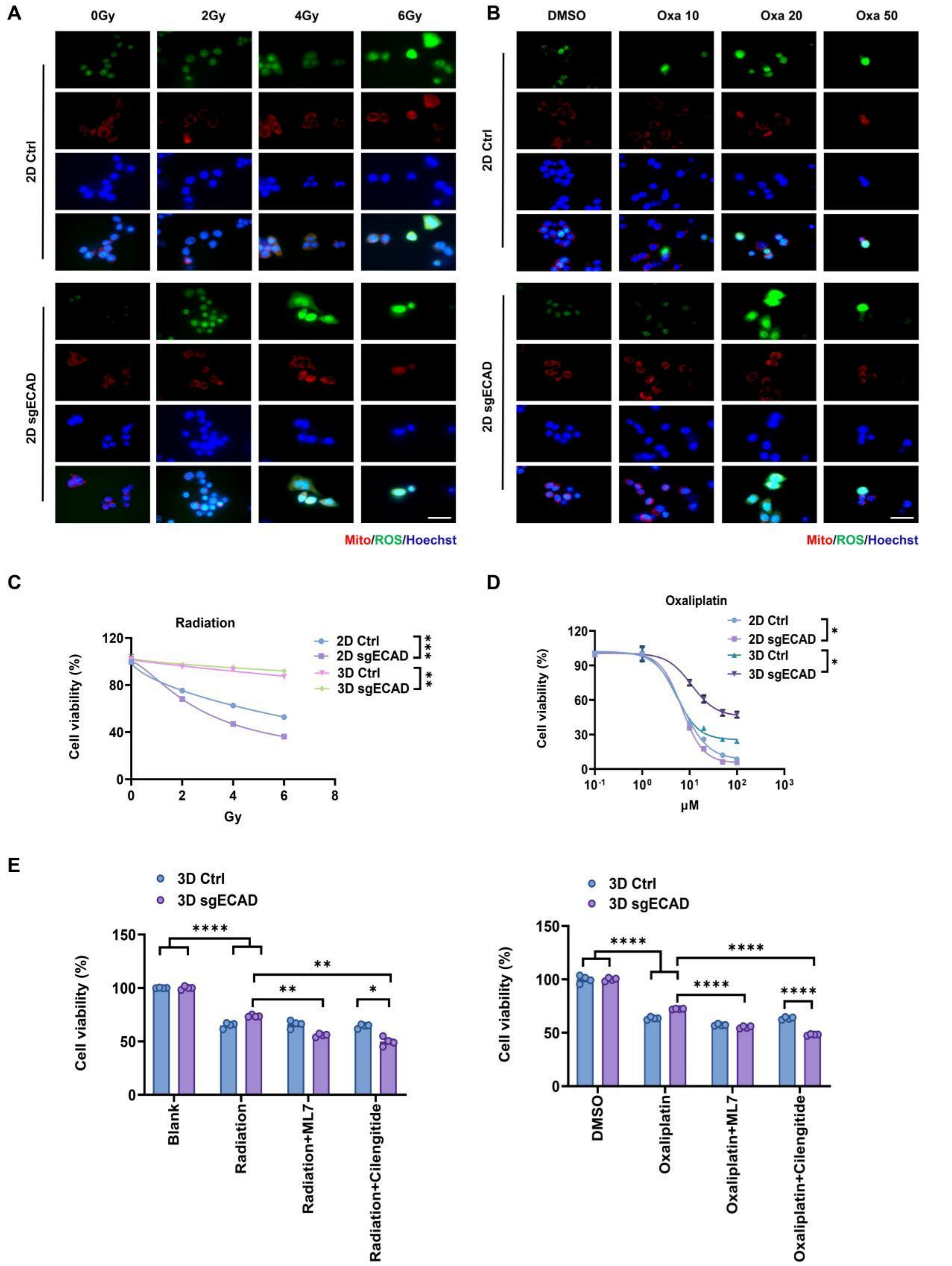

Figure S14 EMT states predict therapeutic resistance of CRC

A. Mitochondrial (**red**) and ROS (**green**) staining of 2D Ctrl and 2D sgECAD cells treated with different doses of radiation (0, 2, 4, 6 Gy) after 24 hours. Nuclei were visualized by Hoechst (**blue**). Scale bar, 100 μm. At least 3 views were randomly

selected for each condition.

B. Mitochondrial (**red**) and ROS (**green**) staining of 2D Ctrl and 2D sgECAD cells treated with DMSO or different concentrations of Oxaliplatin (10, 20, 50 μM) for 48 hours. Scale bar, 100 μm. At least 3 views were randomly selected for each condition.

C. Relative cell viability of 2D Ctrl, 2D sgECAD, 3D Ctrl and 3D sgECAD cells treated with different doses of radiation (0, 2, 4, 6 Gy) after 24 hours. Means ± SEMs; n = 4. $^{**}P < 0.01$, $^{***}P < 0.001$ analyzed by the ordinary one-way ANOVA with Tukey's multiple comparison test.

D. Relative cell viability of 2D Ctrl, 2D sgECAD, 3D Ctrl and 3D sgECAD cells treated with DMSO or different concentrations of Oxaliplatin (0.1, 1, 10, 20, 50, 100 μM) for 48 hours. Means ± SEMs; n = 4. $^{*}P < 0.05$ analyzed by the ordinary one-way ANOVA with Tukey's multiple comparison test.

E. 3D Ctrl and 3D sgECAD cells were pretreated with DMSO, 5 μM ML7, or 5 μM Cilengitide for 6 hours, and then treated with 4 Gy of radiation or 5 μM Oxaliplatin for 24 hours. Relative cell viability of cells was detected. Means ± SEMs; n = 4. $^{*}P < 0.05$, $^{**}P < 0.01$, $^{****}P < 0.0001$ analyzed by the ordinary one-way ANOVA with Tukey's multiple comparison test.

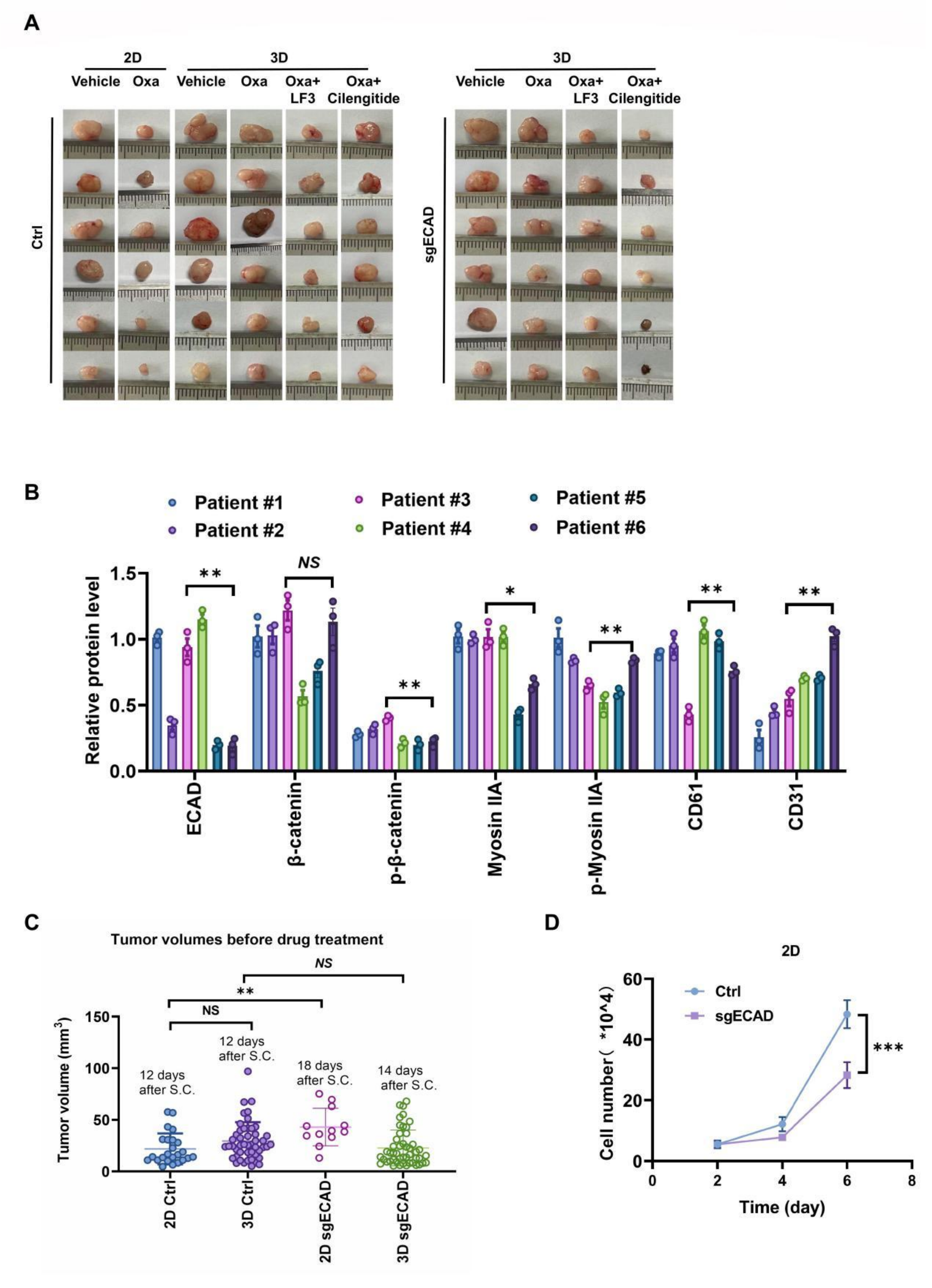

Figure S15 EMT states predict therapeutic resistance of CRC

A. Images of tumor tissue from different mice. Cells were subcutaneously injected into BALB/c nude mice after adjusting the cell concentration to 10^6. Once the tumors reached a diameter of around 5 mm, mice were treated with vehicle, Oxaliplatin (7.5

mg/kg), Oxaliplatin+LF3 (50 mg/kg), or Oxaliplatin+Cilengitide (100 μg) by intraperitoneal injection every two days for 14 days. Six tumors were shown for each group.

B. Proteins were extracted from the tumor tissue of CRC patients. ECAD, β-catenin, phospho-β-catenin, MyosinIIA, phospho-MyosinIIa, CD61, CD31, and TUBULIN protein levels were detected by western blotting. Relative protein levels were normalized to TUBULIN. Means ± SEMs; n = 3. **P* < 0.05, ***P* < 0.01, *****P* < 0.0001 analyzed by the ordinary one-way ANOVA with Tukey's multiple comparison test.

C. Tumor volumes were collected from all the mice at the start of drug injection. Cells were subcutaneously injected (**S.C.**) into BALB/c nude mice after adjusting the cell concentration to 10^6. Drug injections were started when the tumor diameter reached about 5 mm after 12 (2D Ctrl and 3D Ctrl), 14 (3D sgECAD), and 18 (2D sgECAD) days of S.C.. Means ± SEMs; n = 24, 48, 12, 48, respectively. ***NS***, non-significant difference, ***P* < 0.01 analyzed by the ordinary one-way ANOVA with Tukey's multiple comparison test.

D. Ctrl and sgECAD cells were cultured on a 2D 96-well plate for 6 days. Cell numbers were counted every 2 days. Means ± SEMs; n = 3. ****P* < 0.001 analyzed by the ordinary one-way ANOVA with Tukey's multiple comparison test.

Figure 1I

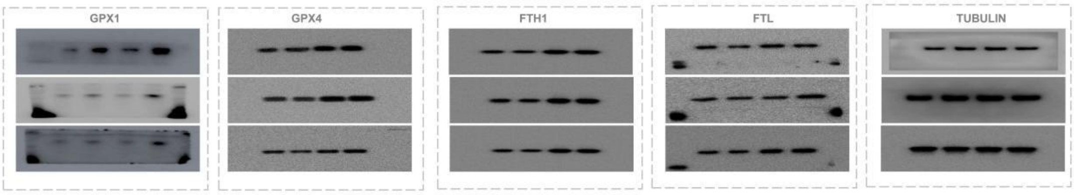

Figure 2B

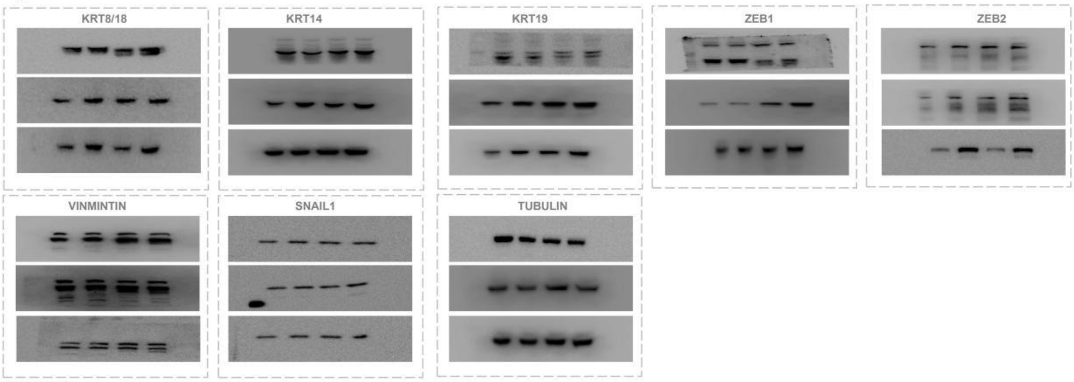

Figure 2G

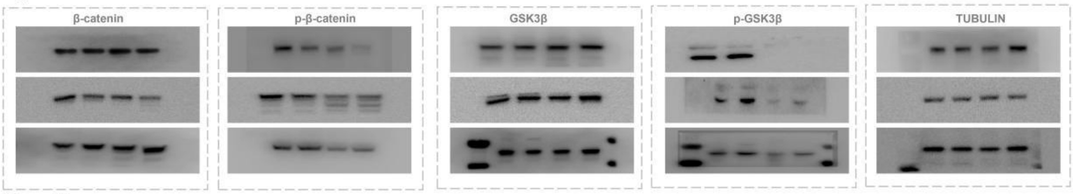

Figure 3A

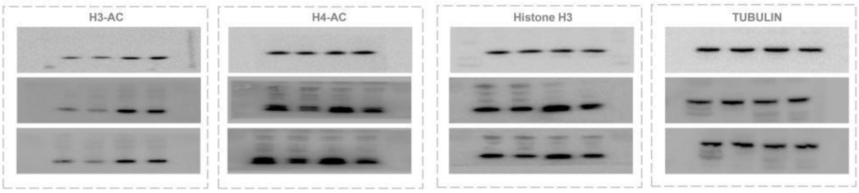

Figure 3K

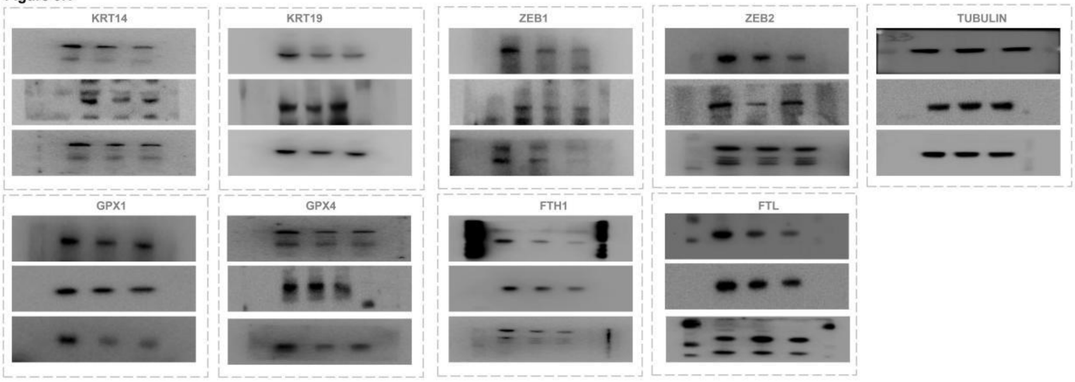

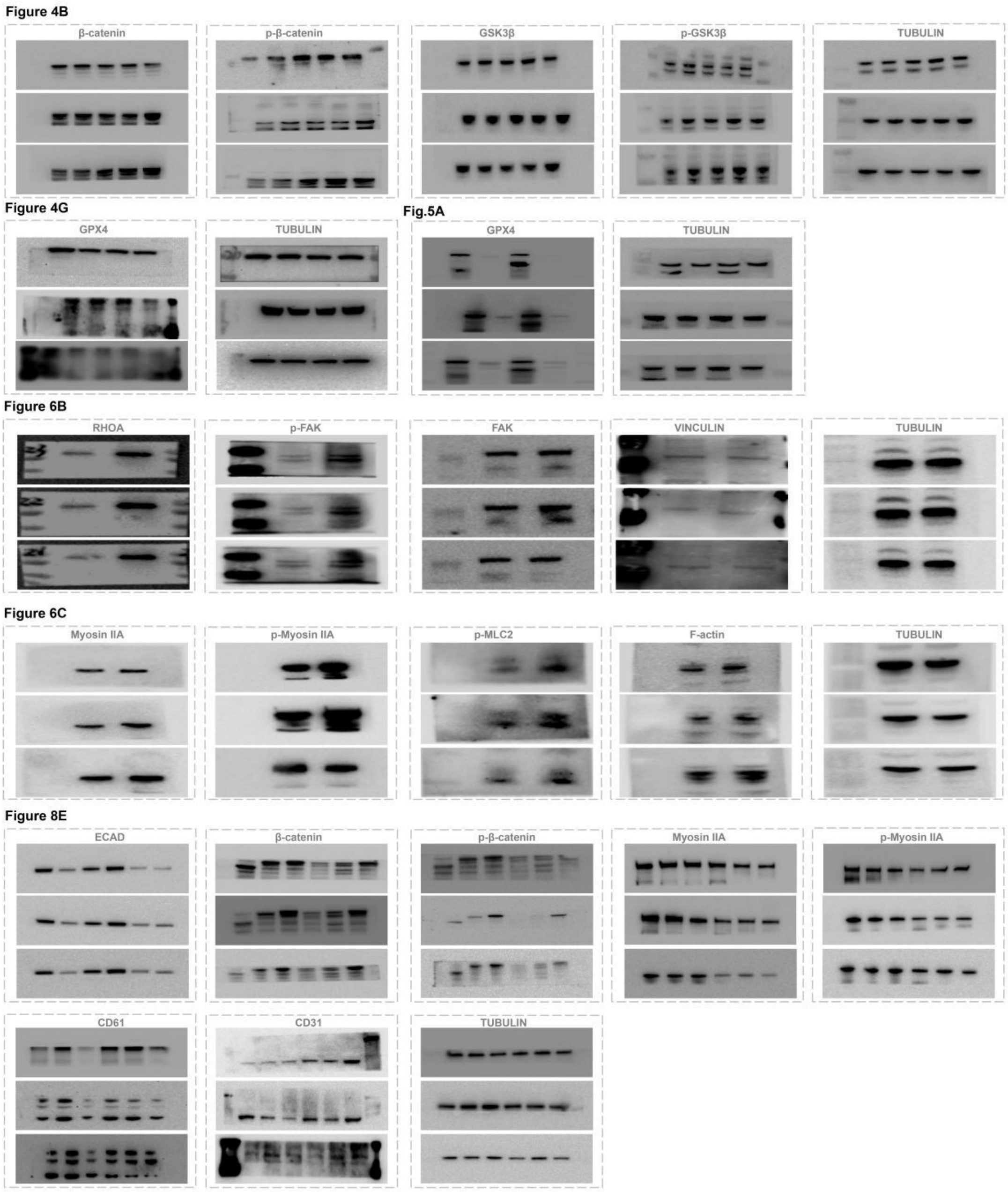

Figure S16 Unmodified western blotting bands for the main figures

Table S1. Patient information.

Table S2. List of antibody information.

Table S3. List of primer sequences for qPCR.

Table S4. List of siRNA and shRNA sequences.

Table S5. Correlation analysis between hybrid EMT markers and ferroptosis regulators.
